# Profiling of open chromatin in developing pig (*Sus scrofa*) muscle to identify regulatory regions

**DOI:** 10.1093/g3journal/jkab424

**Published:** 2021-12-13

**Authors:** Mazdak Salavati, Shernae A Woolley, Yennifer Cortés Araya, Michelle M Halstead, Claire Stenhouse, Martin Johnsson, Cheryl J Ashworth, Alan L Archibald, Francesc X Donadeu, Musa A Hassan, Emily L Clark

**Affiliations:** 1 The Roslin Institute and Royal (Dick) School of Veterinary Studies, University of Edinburgh, Edinburgh EH25 9RG, UK; 2 Centre for Tropical Livestock Genetics and Health (CTLGH), Roslin Institute, University of Edinburgh, Edinburgh EH25 9RG, UK; 3 Department of Animal Science, University of California Davis, Davis, CA 95616, USA; 4 Department of Animal Science, Texas A&M University, College Station, TX 77843, USA; 5 Department of Animal Breeding and Genetics, Swedish University of Agricultural Sciences, Uppsala 750 07, Sweden

**Keywords:** ATAC-Seq, Sscrofa11.1, RNA-Seq, frozen tissue, muscle

## Abstract

There is very little information about how the genome is regulated in domestic pigs (*Sus scrofa*). This lack of knowledge hinders efforts to define and predict the effects of genetic variants in pig breeding programs. To address this knowledge gap, we need to identify regulatory sequences in the pig genome starting with regions of open chromatin. We used the “Improved Protocol for the Assay for Transposase-Accessible Chromatin (Omni-ATAC-Seq)” to identify putative regulatory regions in flash-frozen semitendinosus muscle from 24 male piglets. We collected samples from the smallest-, average-, and largest-sized male piglets from each litter through five developmental time points. Of the 4661 ATAC-Seq peaks identified that represent regions of open chromatin, >50% were within 1 kb of known transcription start sites. Differential read count analysis revealed 377 ATAC-Seq defined genomic regions where chromatin accessibility differed significantly across developmental time points. We found regions of open chromatin associated with downregulation of genes involved in muscle development that were present in small-sized fetal piglets but absent in large-sized fetal piglets at day 90 of gestation. The dataset that we have generated provides a resource for studies of genome regulation in pigs and contributes valuable functional annotation information to filter genetic variants for use in genomic selection in pig breeding programs.

## Introduction

The domestic pig (*Sus scrofa*) is a hugely important farmed animal species globally, contributing a source of healthy animal protein to feed the growing human population. Meeting the increased demand for healthy sustainably produced food from pigs in coming decades will require novel breeding strategies and management practices that will rely on an improved ability to predict phenotype from genotype (Clark *et al.* 2020). High-resolution annotations of the expressed and regulatory regions of farmed animal genomes provide a resource to accurately link genotype to phenotype (Andersson *et al.* 2015). Variants in putative regulatory regions have been associated with >100 phenotypes in humans (Pai *et al.* 2015). Recently, a functional regulatory variant in the gene myosin heavy chain 3 (*MYH3*) was shown to influence muscle fiber type composition in Korean native pigs (Cho *et al.* 2019). There is very little species-specific information about how the genome is regulated in domestic pigs. This lack of knowledge hinders efforts to identify causative variants for complex traits, and a better knowledge of genome regulation might also improve genomic prediction in breeding programs. To address this knowledge gap, we aim to identify regulatory sequences in the pig genome, starting with regions of open chromatin.

Activation of regulatory DNA drives gene expression patterns that influence phenotypic characteristics. Measurement of open chromatin gives a quantitative genome-wide profile of chromatin accessibility appearing as “peaks” in the data generated for each tissue sample (Thurman *et al.* 2012). These peaks can reflect the function of the adjoining regulatory DNA (Thurman *et al.* 2012). The Assay for Transposable Chromatin (ATAC-Seq) (Buenrostro *et al.* 2013, 2015) has been used successfully to profile regions of open chromatin in chicken, cattle, and pig genomes (Halstead *et al.* 2020a, 2020b). In this study, we used the Improved Protocol for the Assay for Transposase-Accessible Chromatin (Omni-ATAC-Seq) (Corces *et al.* 2017) to profile regions of open chromatin in flash-frozen pig muscle tissue samples. The Omni-ATAC-Seq protocol has been used successfully to profile regions of open chromatin in tissue samples from cattle (Alexandre *et al.* 2021).

Muscle is an important tissue in commercial pig production as muscle traits (*e.g.*, meat and carcass quality) act as economic drivers in pig breeding programs. Prior to this study knowledge of open chromatin in pig muscle was limited to data from only two adult animals (Halstead *et al.* 2020b) and four fetuses from three early developmental stages (Yue *et al.* 2021). For this study, we collected semitendinosus muscle tissues from piglets at five different stages of development (three fetal stages, one neonatal, and one juvenile stage). The developmental stages were chosen for their relevance to hyperplasic muscle development in the fetus and postnatal muscle hypertrophy (Ashmore *et al.* 1973; Wigmore and Stickland 1983; Rudar *et al.* 2019). We hypothesized that gene expression and regulation in semitendinosus muscle tissue would change as the piglets aged, allowing us to identify the transcripts and regions of open chromatin that drive myogenesis. Several studies have profiled gene expression during fetal development in pigs (Zhao *et al.* 2011; Yang *et al.* 2015; Zhao *et al.* 2015; Ayuso *et al.* 2016); however, to date only one other study has examined how chromatin openness changes as the piglet develops (Yue *et al.* 2021).

The number of muscle fibers in pigs is proportional to weight at birth (Aiello *et al.* 2018; Stange *et al.* 2020). Low birth weight in pigs has been shown to cause lifelong impairments in muscle development and growth (Rehfeldt and Kuhn 2006). Low birth weight piglets often display “catch up” growth, but at the expense of laying down a higher proportion of body fat compared to normal-sized littermates (Estany *et al.* 2017). Consistent with these observations, mesenchymal stem cells from intrauterine growth-restricted piglets show a differentiation bias toward the adipocyte lineage in comparison with their normal-sized litter mates (Weatherall *et al.* 2020). Low birth weight piglets tend to produce fatter, less valuable carcasses from a production perspective and as such their incidence within pig litters should be kept to a minimum (Pardo *et al.* 2013). Size variation within a litter is likely to be determined by many different physiological variables including variation in placental blood flow (Stenhouse *et al.* 2018) but may also be influenced by genetic and epigenetic factors (Wang *et al.* 2016; Li *et al.* 2020).

The study we present here used samples of muscle tissue from a common commercial breed cross (Large White × Landrace) to generate ATAC-Seq and RNA-Seq data from the same individuals to characterize the expressed and regulatory regions of the genome during development. The aims of the study were to: (1) map regions of open chromatin in semitendinosus muscle tissue from small-, average-, and large-sized male piglets at five developmental stages (days 45, 60, and 90 prenatal and 1 and 6 weeks postnatal) and (2) analyze RNA-Seq data from the same tissues to generate gene expression profiles. The outcomes of the study will help to (1) understand the molecular drivers of muscle growth in pigs; (2) provide a foundation for functionally validating target genomic regions in vitro; and (3) identify high-quality causative variants for muscle growth with the goal of harnessing genetic variation and turning it into sustainable genetic gain in pig breeding programs. The dataset we generate will also provide valuable information for annotating the pig genome, Sscrofa 11.1 (Warr *et al.* 2020), contributing to the efforts of the international Functional Annotation of Animal Genomes (FAANG) consortium to improve the annotation of the reference genomes of farmed animal species (Andersson *et al.* 2015; Giuffra and Tuggle 2019; Clark *et al.* 2020).

## Methods

### Animals

Tissue samples for this study were collected from Large White × Landrace pigs that were euthanized, not specifically for this study but for other on-going projects on the effects of fetal size on pig development at The Roslin Institute. All gilts (*n* = 13) were fed the same diet and kept under one controlled husbandry condition at the research farm. For each of the developmental stages, the number of litters included in the study was as follows: prenatal day 45 = 1 litter, day 60 = 2 litters, day 90 = 5 litters, and postnatal 1 week = 2 litters and 6 weeks = 3 litters. Gilt identifier numbers for each litter are included in Table 1. Artificial insemination with semen from two unrelated sires was used to generate the fetuses for both pre- and postnatal time points. Fetal tissues were collected after the pregnant sow was euthanized with sodium pentobarbitone 20% w/v (Henry Schein Animal Health, Dumfries, UK) at a dose of 0.4 ml/kg by intravenous injection. Postnatal samples were collected after euthanasia by captive bolt. Tissues from the 1-week-old piglets were collected preweaning and from the 6-week-old piglets postweaning. Six-week-old piglets post weaning were fed a standard concentrate ration and kept under routine husbandry conditions.

**Table 1:** Details of hind leg muscle tissues sampled, for ATAC-Seq and RNA-Seq, from piglets at five developmental stages

Sample ID	Gilt identifier	Age in days	Muscle tissue sampled	Piglet size	ATAC-Seq	RNA-Seq
Prenatal time points						
D45L220716	23743	45	Whole hind leg	Largest	Yes	Yes
D45N220716	23743	45	Whole hind leg	Average	Yes	Yes
D45S220716	23743	45	Whole hind leg	Smallest	Yes	Yes
D60L120916	23982	60	Semitendinosus	Largest	Yes	Yes
D60N120916	23982	60	Semitendinosus	Average	Yes	Yes
D60S120916	23982	60	Semitendinosus	Smallest	Yes	No
D60S23976	23976	60	Semitendinosus	Smallest	No	Yes
D90L251016	23956	90	Semitendinosus	Largest	Yes	Yes
D90L111016	23964	90	Semitendinosus	Largest	Yes	Yes
D90L121016	23963	90	Semitendinosus	Largest	Yes	Yes
D90N251016	23956	90	Semitendinosus	Average	Yes	Yes
D90N111016	23964	90	Semitendinosus	Average	Yes	Yes
D90N031115	87480	90	Semitendinosus	Average	Yes	Yes
D90N121016	23963	90	Semitendinosus	Average	Yes	Yes
D90N231115	87502	90	Semitendinosus	Average	Yes	Yes
D90S231115	87502	90	Semitendinosus	Smallest	Yes	Yes
D90S251016	23956	90	Semitendinosus	Smallest	Yes	Yes
D90S111016	23964	90	Semitendinosus	Smallest	Yes	Yes
D90S121016	23963	90	Semitendinosus	Smallest	Yes	No
		**Age in weeks**				
Postnatal time points						
1WKL100918	21	1	Semitendinosus	Largest	Yes	Yes
1WKS100918	22	1	Semitendinosus	Smallest	Yes	Yes
6WKA050219	6455	6	Semitendinosus	Average	Yes	Yes
6WKS050219	6432	6	Semitendinosus	Smallest	Yes	Yes
6WKS131218	6383	6	Semitendinosus	Smallest	No	Yes
6WKA131218	6383	6	Semitendinosus	Average	No	Yes
6WKL131218	6383	6	Semitendinosus	Largest	No	Yes
Cryopreserved nuclei preparations						
6WKA050219CN	6455	6	Semitendinosus	Average	Yes	No
6WKS050219CN	6432	6	Semitendinosus	Smallest	Yes	No

### Sample collection of frozen muscle tissue samples for ATAC-Seq and RNA-Seq

The tissue samples used for this study were from archived material (with the exception of the piglets that were 6 weeks of age) collected from the largest-, smallest-, and average-sized male piglets per litter at five different developmental stages (Table 1). The largest-, smallest-, and average-sized piglets from each litter were selected according to body weight at the time of sampling for the fetal time points and birth weight for the postnatal time points (Supplementary Table S1). Developmental stages were chosen, according to previous studies (Ashmore *et al.* 1973; Wigmore and Stickland 1983; Rudar *et al.* 2019) as follows:

Day 45 of gestation—when primary muscle fibers form.

Day 60 of gestation—when secondary muscle fibers begin to form.

Day 90 of gestation—when fiber formation ceases after which subsequent muscle growth occurs through fiber hypertrophy.

One week of age—during active muscle hypertrophy.

Six weeks of age—once muscle hypertrophy has leveled off.

**Figure 1:**
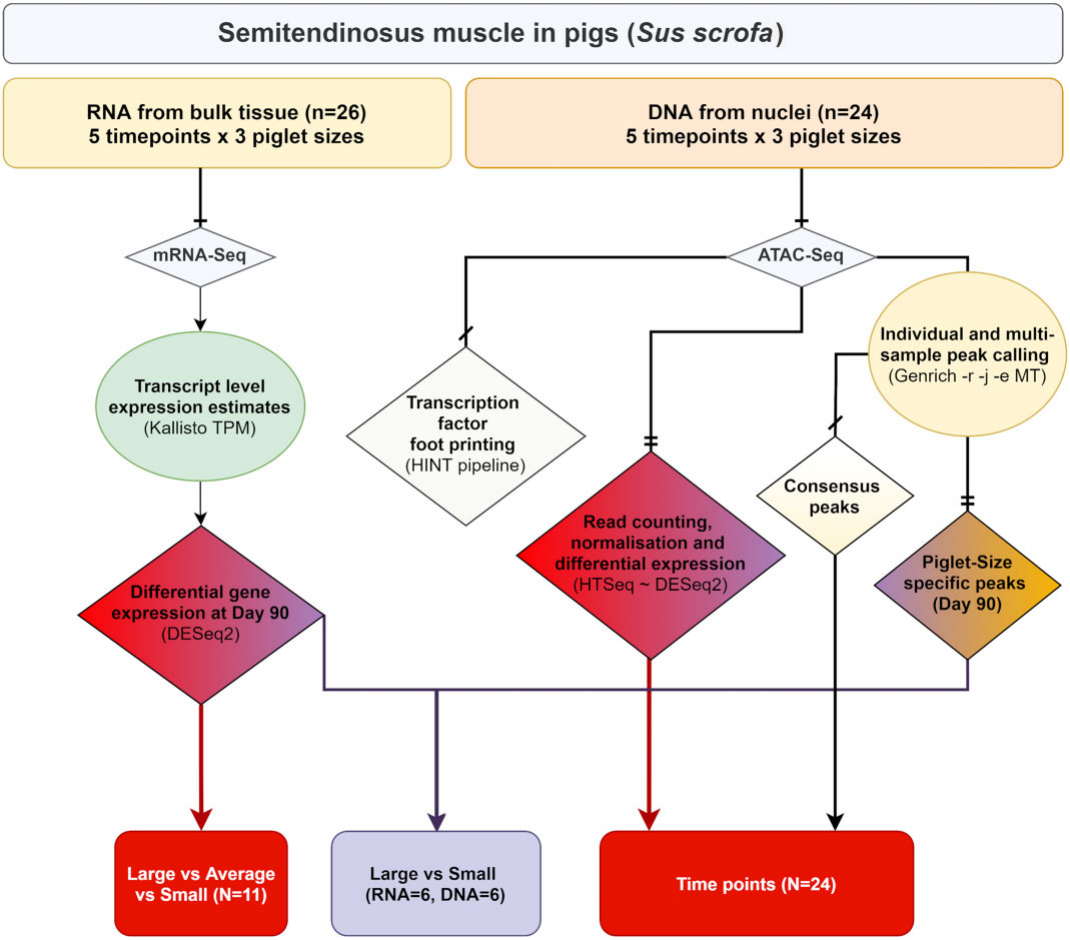
Flow chart describing the experimental design and samples included in each stage of the analysis performed in this study. Data were analyzed in two streams: (1) analysis of gene expression using the mRNA-Seq data from isolated RNA, and (2) analysis of peaks of open chromatin using the ATAC-Seq data from isolated tagmented nuclear DNA. Color coding indicates where there are overlaps in the analysis performed for each component of the study.

Due to limited sample availability, the experimental design is unbalanced (Table 1). Only one complete set of littermates (smallest, average, and largest) was included in the analysis for the day 45 (*n* = 3) and 60 (*n* = 3) time points. For the 6 weeks of age time point (*n* = 5), one large sample was unavailable for inclusion in the analysis. At day 90 of gestation (*n* = 11) three complete litters and one incomplete litter were included, while at 1 week of age samples were only available from the smallest and largest piglets from one litter (*n* = 2). We have included a flow chart describing which samples were analyzed at each stage of this study (Figure 1).

Samples were collected from the semitendinosus muscle from the hind leg of piglets from each developmental stage (Table 1). The only exception was day 45, when whole hind leg muscle tissue was collected, because it was not possible to differentiate specific muscle types at this early stage of development. Each sample was flash frozen in liquid nitrogen, as quickly as possible within an hour post-euthanasia and stored at −80°C for future analysis. From the 6-week-old piglets, additional samples were collected in sucrose buffer to isolate and cryopreserve nuclei according to the method described in (Halstead *et al*. 2020a). Cryopreserving isolated nuclei for a small number of samples would allow us to validate the data we generated from the flash-frozen material, which we were optimizing for muscle tissue. The protocol for collection of tissue samples is available via the FAANG Data Coordination Centre (FAANG-SOP1 2021).

### Isolation of cryopreserved nuclei from fresh muscle tissue and preparation of tagmented nuclear DNA

We used the protocol described in Halstead *et al.* (2020a) to isolate and cryopreserve intact nuclei from fresh muscle tissue samples from the 6-week-old piglets (Table 1). Briefly, each tissue sample was transferred to a GentleMACS C-tube (Mitenyi Biotec, Germany) with sucrose buffer and homogenized. The homogenate was then filtered and Dimethyl Sulfoxide (Sigma Aldrich, USA) added (10% final concentration), before freezing at −80°C overnight in a Mr Frosty (Nalgene, USA), and then transferring to a −80°C freezer for long-term storage. The full protocol for preparation of cryopreserved nuclei from fresh muscle tissue is available via the FAANG Data Coordination Centre (FAANG-SOP2 2021).

To prepare tagmented DNA the cryopreserved nuclei preparations were thawed slowly at room temperature by adding 500 µl of cold 1× phosphate-buffered saline (PBS), filtered using a 70 µm corning cell strainer (Sigma Aldrich, USA) then the filtrate (containing the nuclei) centrifuged at 500*g* at 4°C in a swinging bucket centrifuge for 10 min. After centrifugation, the pellet was resuspended in 1 ml cold ATAC-Seq RSB Buffer + 0.1% Tween 20 (Sigma Aldrich, USA) for lysis and centrifuged for 10 min at 500*g* at 4°C. The pellet of nuclei was then washed in PBS and resuspended in 50 µl transposition mix (25 µl TD buffer, 2.5 µl TDE1 enzyme, Molecular Biology Grade Sterile H_2_O) from the Nextera DNA Sample Prep Kit (Ilumina, USA). The pellet was incubated with the transposition mix for 60 min at 37°C at 300 rpm on a thermomixer. The pellet of transposed nuclear DNA was purified with a MinElute PCR purification kit (Qiagen, Germany), eluted in 15 µl of Buffer EB and stored at −20°C. The full protocol is available via the FAANG Data Coordination Centre (FAANG-SOP3 2021).

### Isolation of nuclei from frozen muscle tissue and preparation of tagmented nuclear DNA

ATAC-Seq libraries were prepared using a version of the Omni-ATAC-Seq protocol (Corces *et al.* 2017). Some optimization of the protocol for flash-frozen pig muscle tissue samples was required for this study. The main modification that we introduced to the protocol was an initial dissociation step using a GentleMACS Dissociator (Mitenyi Biotec, Germany), essentially combining the Omni-ATAC-Seq protocol with the initial steps from Halstead *et al.* (2020a). The protocol is described in full in FAANG-SOP4 (2021) and summarized here. The components of each of the buffers are included in Supplementary Table S2. Each flash-frozen tissue sample (∼200 mg per sample) was chopped into small pieces over dry ice and then dissociated in a GentleMACS C-tube (Mitenyi Biotec, Germany) in 1 ml of 1× HB buffer (+Protease Inhibitor Cocktail). The samples were dissociated using program m_muscle_0.1_0.1 (equivalent to “E0.1c Tube”) twice on a GentleMACS Dissociator (Mitenyi Biotec, Germany). Immediately after dissociation, the samples were filtered through a 70-μm corning cell strainer (Sigma Aldrich, USA) then the filtrate centrifuged at 3000*g* for 5 min. The pellet was resuspended in 400 μl 1×HB buffer and transferred to a 2 ml Eppendorf Protein Lo-Bind tube (Eppendorf, UK). Four-hundred microliter of 50% Iodixanol solution (Opti-Prep Density Gradient Medium; SLS, UK) was added to the 400 μl of cell solution (final 25% Iodixanol). An Iodixanol gradient was then created and samples transferred to a swinging bucket centrifuge and spun for 25 min at maximum speed at 4°C with no brake. A thin “whitish” band appeared between layers two and three of the gradient. Evaluation and counting of nuclei was performed by staining with Trypan Blue (Thermo Fisher Scientific, USA). One milliliter of ATAC-RSB Buffer + 0.1% Tween 20 was then added to lyse the nuclei and the sample centrifuged for 10 min at 500*g* at 4°C. The pellet was then gently resuspended in 50 µl transposition mix for tagmentation as described for cryopreserved nuclei samples above.

### ATAC-Seq library preparation

The library preparation protocol, adapted from Corces *et al.* (2017), was used for the flash-frozen tissues and the cryopreserved nuclei preparations. The protocol described in full is available via the FAANG Data Coordination Centre (FAANG-SOP5 2021). A PCR mix was set up comprising 10 μl molecular biology grade H_2_O, 2.5 µl Ad1 primer 25 µM, 2.5 µl Ad2.x primer 25 µM (variable index see Supplementary Table S3), and 25 µl 2× NEBNext Hi-Fi PCR mix (NEB, USA) per reaction. Ten microliters of transposed DNA was added to each reaction and five amplification cycles of the following PCR performed: 72°C for 5 min, 98°C for 30 s, 98°C for 10 s, 63°C for 30 s, 72°C for 1 min. The GreenLeaf Quantitative PCR Protocol (Buenrostro *et al.* 2015) was used to determine the number of additional PCR amplification cycles that were required for each sample, to stop amplification prior to saturation and avoid variation across samples caused by PCR bias. Samples for which more than 5–7 additional cycles were required were discarded due to the high probability of PCR bias caused by additional cycles. Amplified ATAC-Seq libraries were then purified with a MinElute PCR purification kit (Qiagen, Germany). Library quality was checked on the Agilent 2200 TapeStation System (Agilent Genomics, USA). Libraries were assessed for quality according to an even distribution of fragments and a clearly differentiated sub-nucleosomal fragment as described in Halstead *et al.* (2020a). If library quality was sufficient the sub-nucleosomal fragment (150–250 bp) was size selected, to minimize the signal to noise ratio, as suggested in Halstead *et al.* (2020a). Size selection was performed using a Thermo Scientific E-Gel System (Thermo Fisher Scientific, USA). To check the size of the selected fragment, an aliquot was run on the Agilent 2200 TapeStation System (Agilent Genomics, USA). After size selection, the libraries were pooled and stored at −20°C prior to sequencing.

### Sequencing of ATAC-Seq libraries

Pooled libraries (four batches) were sequenced to generate 50-nt paired-end reads on an Illumina NovaSeq 6000 platform using a single S2 flow cell. All of the libraries generated >90 M paired-end reads (min: 9.8e+07, max: 3.5e+08, median: 1.97e+08).

### ATAC-Seq data processing and mapping

The quality of the raw sequence data was evaluated using FastQC v0.11.9 (Andrews 2010) and multiQC v1.9 (Ewels *et al.* 2016). The paired-end reads were trimmed using Trimmomatic v0.39 (*ILLUMINACLIP:Trimmomatic-0.39/adapters/NexteraPE-PE.fa: 2:30:10:1:true SLIDINGWINDOW:5:20 MINLEN:30*; Bolger *et al.* 2014). The trimmed reads were then mapped to the Sscrofa11.1 pig reference genome (Warr *et al.* 2020) available from Ensembl (GCA_000003025.6) using Bowtie2 v2.3.5.1 and the default flags of the*—very-sensitive* mode followed by excluding unmapped reads and marking PCR duplicates. PCR duplicates were marked using PicardTools v2.23.0 (Li *et al.* 2009; Broad Institute 2019). The BAM files that were generated were then sorted and indexed using samtools v1.6 (Li 2011). Overall on average more than 75 M reads per sample were uniquely mapped (min: 2.47e+07, max: 1.28e+08, median: 7.74e+07, mean ± SD: 7.72e+07 ± 3.15e+07). The PCR duplication level (post-mapping) was 43% ± 8 (mean ± SD) across all libraries.

The following parameters were measured as recommended by the ENCODE project for the validation of ATAC-Seq libraries (Davie *et al.* 2015, 2018): fragment size distribution [PicardTools v2.25.4 (Broad Institute 2019)], Fragment in peak [FRiP score, deepTools v3.5.1 (Ramírez *et al.* 2016)], transcription start site enrichment score [TSS-ES ATAC-SeqQC v1.14.4 (Ou *et al.* 2018)], nonredundant fraction [NRF samtools v1.6 (Li *et al.* 2009)], nucleosome-free region score [NFR score ATAC-SeqQC v1.14.4 (Ou *et al.* 2018)], and PCR bottlenecking coefficient [PBC ATAC-SeqQC v1.14.4 (Ou *et al.* 2018)]. A detailed table of QC scores is included in Supplementary Table S6. Annotation and visualization of the annotated peaks were carried out using ChIPseeker v1.28.3 package in R v>4.0.0 (Yu *et al.* 2015; R Core Team 2017).

### ATAC-Seq peak calling using Genrich

ATAC-Seq peak calling was performed using Genrich v0.5 (Gaspar 2020) under the ATAC-Seq mode while excluding PCR duplicates and mitochondrial reads (used flags: *-r -j -e MT*). Two rounds of peak calling were performed as follows: (1) peak calling on individual samples (*n* = 24) and (2) aggregated multi-sample peak calling for each piglet size. All the scripts used for this analysis are found in Supplementary File S1 and the code repository (Salavati 2021).

### Differential peak analysis based on read counts

A consensus set of ATAC-Seq peaks was created for the purpose of differential peak analysis. The consensus set, from individual sets that were called in all 24 samples, was created using bedtools v2.26.0 (*bedtools merge -i all_samples.bed -d10 -c 4,7,10,4 -o count_distinct*,*mean*,*mode*,*distinct*). Peaks that were within ≤10 nucleotides of another peak were merged into one peak, and a support value (*i.e.*, the number of tissue samples in which the peak was present) was calculated for each peak. Peaks with a support value of <3 (*i.e.*, they were present in <3 tissue samples) were removed, resulting in a total of 12,090 ATAC-Seq peaks, which will now be referred to as the “consensus set.” A read fragment filtering and analysis workflow was devised similar to a recently published framework by Yan *et al.* (2020). Briefly, the mapped BAM files were filtered for high mapping quality, nonPCR duplicates and nonmitochondrial reads using samtools v1.6 (*samtools view -h -f2 -q10 -F1548 -bS*). A read count for each sample (using high-quality BAM files) was then generated using ht-seq v0.13.5 (Anders *et al.* 2015) against the consensus set of ATAC-Seq peaks (*htseq-count--stranded=no –type=region*). The library size for each BAM file was then used to normalize the read counts for multidimensional scaling analysis as described by Yan *et al.* (2020) using the following equation:

$$\mathrm{Normalized counts}=\log2\left(\left(\frac{\mathrm{raw counts}}{\mathrm{library size}}\times 1.0+E08\right)+1\right)$$

DESeq2 v1.30.1 (Love *et al.* 2014) was used for differential peak analysis, of the raw read counts, to compare across developmental time points. A Likelihood Ratio Test (reduced model) was used for the analysis of the time points (design: ∼ size + time; reduced: ∼ size). A multiple testing *P*-value correction was performed using the Benjamini–Hochberg (1995) method and a 10% false discovery rate (FDR) was considered as the threshold of significance.

### Multidimensional scaling analysis for comparison of ATAC-Seq libraries

Nonlinear multidimensional scaling (NMDS) of the ATAC-Seq libraries was performed using the MASS::IsoMDS package (Venables and Ripley 2002) to ensure that there were no obvious outlying samples and that tissues of the same type clustered together in a biologically meaningful manner. A distance matrix (Manhattan distance) was produced using the consensus set of ATAC-Seq peaks and the normalized read counts as described in the previous section. The distance matrix was then processed for multidimensional scaling. For data validation purposes, NMDS was also used to compare the ATAC-Seq libraries prepared from either flash-frozen muscle tissue or cryopreserved nuclei from two 6-week-old piglets.

### Transcription factor footprint analysis

The HMM-based Identification of Transcription factor footprints (HINT) pipeline from the Regulatory Genomics Toolbox (RGT; v0.12.3; Li *et al.* 2019) was used to compare transcription factor (TF) activity between developmental stages or piglet sizes. For a given comparison, the rgt-hint command in foot printing mode was used to identify TF footprints within peaks based on ATAC-Seq signal in each condition. When comparing consecutive developmental stages, the ATAC-Seq peaks identified for each stage were merged with Bedtools (v2.26.0), and footprints were identified within the merged set. When comparing different piglet sizes within a developmental time point, footprints were identified within the peak set for that time point (regardless of piglet size). ATAC-Seq signal for a given condition included aligned reads from all biological replicates (excluding libraries from cryopreserved nuclei), which were combined and filtered to remove duplicates using Samtools (v1.7). Footprints were matched to known motifs in JASPAR (Fornes *et al.* 2020) with rgt-motif analysis, and rgt-hint in differential mode was then used to compare the activity of each TF between two given conditions using bias-corrected signal.

### RNA isolation and quality control

The RNA isolation protocol is described in full in FAANG-SOP6 (2021). RNA was extracted from approximately 60 mg of tissue. Tissue samples were homogenized in 1 ml of TRIzol (Thermo Fisher Scientific, USA) with CK14 (VWR, USA) tissue homogenizing ceramic beads on a Precellys Tissue Homogeniser (Bertin Instruments; France) at 5000 rpm for 20 s. RNA was then isolated using the TRIzol protocol and column-purified to remove DNA and trace phenol using an RNeasy Mini Kit (Qiagen, Germany) following the manufacturer’s instructions. RNA integrity (RIN^e^) was estimated on an Agilent 2200 TapeStation System (Agilent, USA) to ensure that RNA quality was of RIN^e^ >7. RIN^e^ and other quality control metrics for the RNA samples are included in Supplementary Table S4.

### Poly-A-enriched library preparation and sequencing

Strand-specific paired-end reads with a fragment length of 100 bp for each sample were generated by Edinburgh Genomics, using the Illumina TruSeq mRNA library preparation protocol (poly-A selected; Illumina; Part: 15031047 Revision E). mRNA-Seq libraries were sequenced on an Illumina NovaSeq 6000 platform to generate >66 M paired-end reads per sample (min: 6.6e+07, max: 1.21e+08, mean: 9.17+e07).

### RNA-Seq data analysis workflow

The raw sequence data were quality-controlled and trimmed using Trimmomatic (Bolger *et al.* 2014). The Kallisto aligner (Bray *et al.* 2016) was used for expression quantification of the RNA-Seq data. Briefly, a reference transcriptome fasta file of coding sequences was obtained from Sscrofa11.1 Ensembl v100 to build a Kallisto index file using default settings. The trimmed reads were then mapped for transcript-level expression quantification (*de novo*) in kallisto with*—bias* option activated. The output tab-separated value files were then imported to R using txImport package (Soneson *et al.* 2016) for further analysis and visualizations.

The transcript per million mapped (TPM) expression estimates for each sample were investigated using principal component analysis (PCA) in FactoMineR to identify any spurious samples that did not cluster as expected (Luo *et al.* 2009). Differential expression (DE) analysis was performed only on the three sizes of fetal piglet (small, average, and large) at day 90 of gestation. The Likelihood Ratio Test (LRT) model of DESeq2, including post hoc analysis, was used with small size as the reference level, *i.e.*, denominator in log2 fold change (log2FC) [*DESeqDataSetFromTximport(txi = dds, design = ∼ Piglet size)*]. After multiple correction of *P*-values using the BH method (Benjamini and Hochberg 1995), an FDR of 10% was considered as the significance threshold.

### Overlay of differentially expressed genes and ATAC-Seq peaks

For the day 90 samples only, we reanalyzed the ATAC-Seq peaks in the three sizes of fetal piglet per litter (large, average, and small). Peak calling was performed using the same Genrich flags as previously described (aggregated multi-sample method), and we separated peaks shared between all size classes and size class-specific peaks with bedtools v2.26.0 (Quinlan and Hall 2010). The size-specific peaks generated for the fetal piglets at day 90 of gestation and the scripts used to produce them are also found in Supplementary File S1 and the code repository (Salavati 2021).

An overlay of genes that were differentially expressed between the large- and small-sized fetal piglets at day 90 was performed using ATAC-Seq peaks within 10-kb vicinity of the differentially expressed genes (either upstream or downstream). This overlay would show us which of the differentially expressed genes had an ATAC-Seq peak in their vicinity and whether that peak was present in both large- and small-sized fetal piglets, or only in one of the two sizes. The distance from the start of the gene model to the start of the ATAC-Seq peak was used as a coordinate system (*i.e.*, positive values meant that the peak was either within the gene or within the 5′ 10-kb upstream region of the gene, and negative values corresponded to 10 kb from the 3′ end of the gene).

### Statistical analysis software and packages

All data analysis for this study was performed via bash scripting and use of R (R Core Team 2017) on the University of Edinburgh research computing facility (Edinburgh 2020). The data analysis protocol for ATAC-Seq and RNA-Seq is available in FAANG-SOP7 (2021) and FAANG-SOP8 (2021).

## Results

### ATAC-Seq data from frozen pig muscle tissues

**Figure 2:**
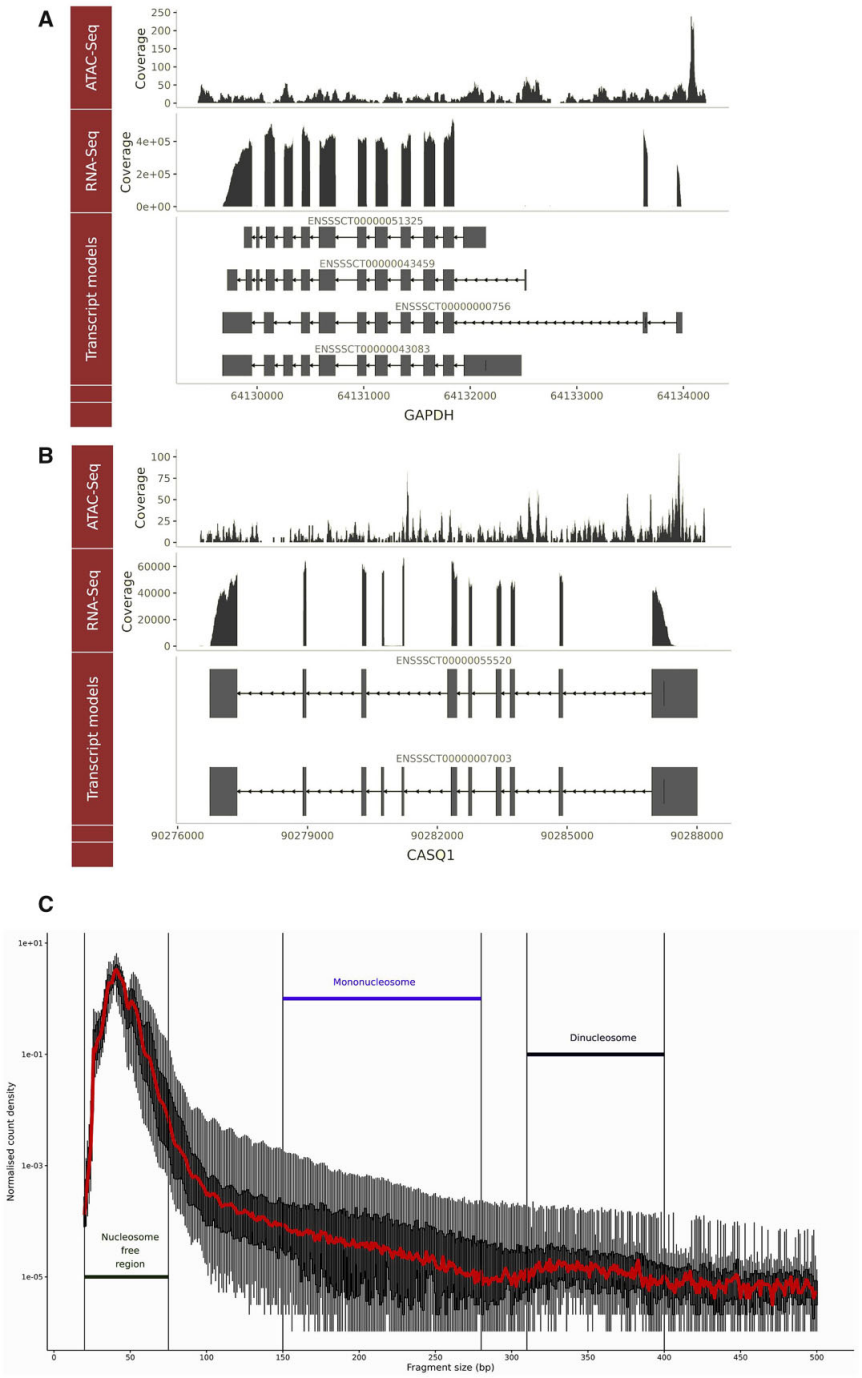
Genomic track visualization of the ATAC-Seq and RNA-Seq datasets by the presence of a signal at the genomic coordinates of two genes *GAPDH* and *CASQ1*. The raw ATAC-Seq read counts and RNA-Seq TPM counts, from a representative with a library size closest to the average for the set, are shown in tracks above the transcript model for each gene. Two genes are shown: (A) *GAPDH* a housekeeping gene, and (B) *CASQ1* a gene involved in muscle growth and development. (C) The fragment size distribution of all 24 libraries is plotted against normalized read count density [log2(count/max(count))]. Fragment size distribution was calculated after removing PCR duplicates, multi-mapped reads, improper pairs or mapping quality <30.

ATAC-Seq libraries from four different batches (24 samples in total) were multiplexed and sequenced to achieve 2.02e+08 average reads per sample (min: 9.8e+07, max: 3.5e+08, median: 1.97e+08). Reads were evenly distributed between barcodes across the first three batches. The fourth batch, which included only two samples that had a higher concentration of starting DNA, resulting in more reads per sequencing run compared to the other 22 samples (details in Supplementary File S2). Average chromosomal coverage across autosomes was 7.2×, 4× for X, 3.2× for Y, and 4.49e+04 for the mitochondrial chromosome (Supplementary Figure S1). Visual comparison of the ATAC-Seq and the RNA-Seq reads, mapped to the Sscrofa11.1 genome, was used to check for consistency between the two datasets. For example, Figure 2 shows the ATAC-Seq and RNA-Seq data as parallel tracks for a housekeeping gene (*GAPDH*) (Figure 2A), and a gene related to muscle development (*CASQ1*) (Figure 2B). Read coverage of all 24 ATAC-Seq samples and the consensus peak called for *GAPDH* has also been visualized in Supplementary Figure S2.

We also measured the fragment size distribution of the 24 ATAC-Seq samples, which showed that read density was highest at the putative nucleosome-free region (approximately 50 bp insert size) followed by the mononucleosome region (∼150–200 bp) and, then, the dinucleosome (∼300–400 bp) region as shown in Figure 2C.

### Multidimensional scaling analysis of the ATAC-Seq libraries from frozen tissue samples

**Figure 3:**
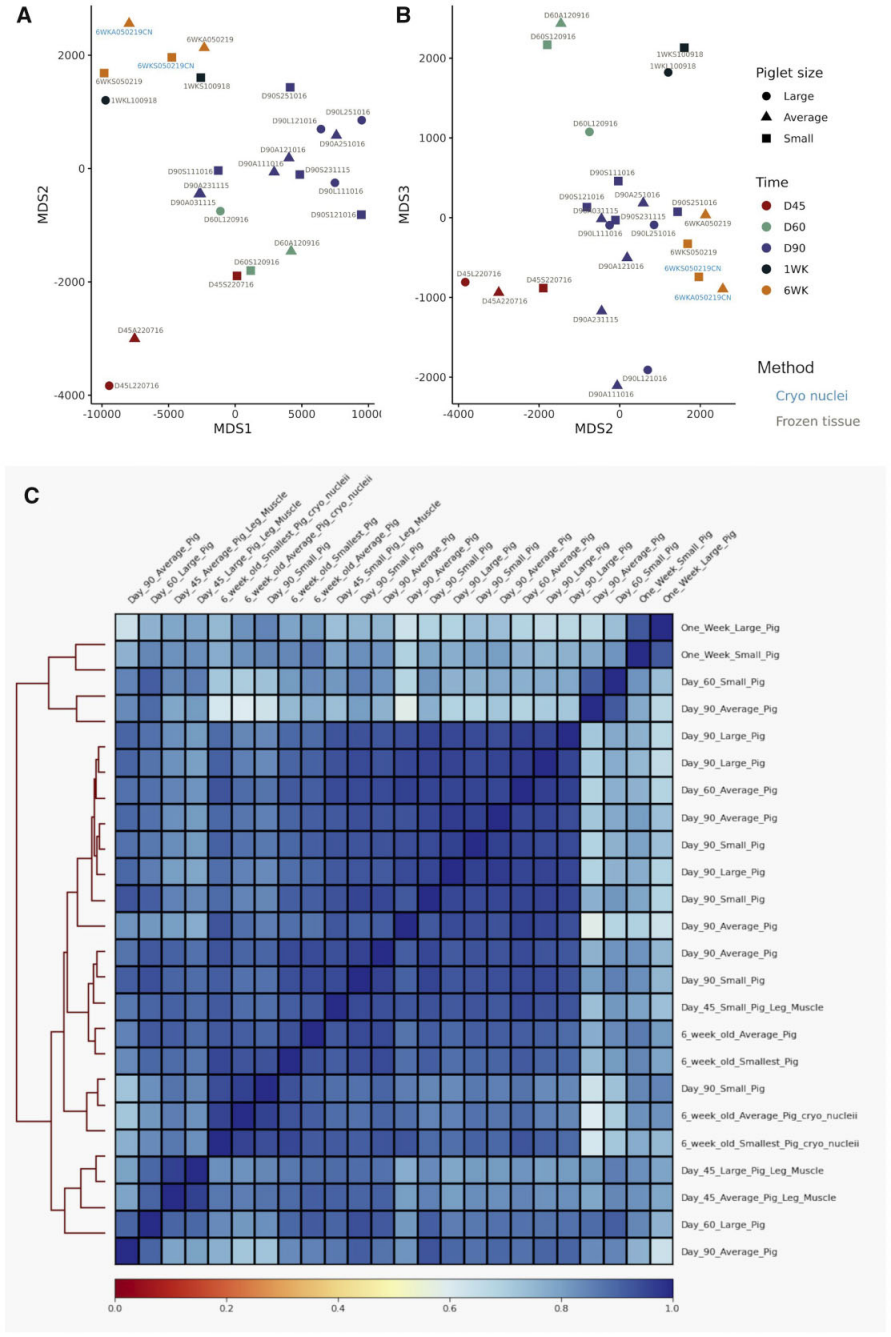
NMDS and correlation analysis of the ATAC-Seq open chromatin consensus set in all samples. (A, B) Dimension reduction plot of the ATAC-Seq dataset based on the NMDS analysis components 1–3. The normalized read count for each sample [as described in Yan *et al.* (2020)] was used for the input matrix for NMDS. Component 1 *vs* 2 (MDS1 *x*-axis; MDS2 *y*-axis) is shown in (A) and component 2 *vs* 3 (MDS2 *x*-axis; MDS3 *y*-axis) in (B). Samples from different developmental time points are indicated by color and the piglet size group by shape. Label colors are used to differentiate between libraries prepared from cryopreserved nuclei and frozen tissue. (C) Correlation heatmap of all 24 samples based on deepTools v3.5.1 binned read coverage (multiBamSummary). A correlation matrix calculated by deeptools was plotted with samples on the top and right hand side along with a hierarchical clustering dendrogram on the left hand side. The sample pairwise correlation coefficient value was used as the color scale of the heatmap (0 = red, 1 = blue).

NMDS was used to ensure that the ATAC-Seq dataset was biologically meaningful, reproducible and there were no outlying samples (*i.e.*, samples from the same developmental stage should have a similar peak distribution and cluster together). NMDS was performed using the consensus set of peaks and the normalized ATAC-Seq read counts. The input matrix, which was converted to a Manhattan distance matrix prior to analysis, consisted of 12,090 consensus peaks and 24 samples. The sample separation on the first two components of the NMDS was driven by the developmental time points. The second and third components of the plot showed the greatest segregation between the developmental time points as shown in Figure 3A (NMDS components 1 and 2) and Figure 3B (NMDS components 2 and 3). The cryopreserved nuclei samples are included for comparison with the ATAC-Seq libraries prepared from flash-frozen muscle tissue. As an additional validation of the dataset, we expected these samples to cluster by developmental stage rather than nuclei isolation method, which was the case (Figure 3, A and B). A correlation heatmap of the 24 ATAC-Seq samples also indicated that the samples for each time point clustered closely together (Figure 3C). The heatmap input matrix was based on the deepTools v3.5.1 multiBamSummary binned (100 bp) read coverage.

### Multidimensional scaling analysis of ATAC-Seq libraries prepared from either flash-frozen muscle tissue or cryopreserved nuclei

To validate the results from the Omni-ATAC-Seq protocol NMDS was also used to compare ATAC-Seq libraries prepared from either flash-frozen muscle tissue or cryopreserved nuclei from two 6-week-old piglets. The two libraries prepared for the cryo-preserved nuclei samples clustered closely with the libraries prepared for flash-frozen tissue indicating that there was little difference in the data generated by the two protocols (Figure 3). Other metrics, including the percentage of ATAC-Seq peaks within promoter, proximal, distal regions, or within a gene model were also used to compare the libraries prepared from cryopreserved nuclei and flash-frozen tissue and showed little differences between the libraries (Supplementary Figure S3). There were no statistically significant differences detected between the two protocols for any of the ATAC-Seq quality control metrics chosen (ANOVA; *P* > 0.05) except the TSS enrichment score (TSS-ES; *t*-test *P* = 0.045; cryo nuclei *vs* frozen tissue mean TSS-ES 8.5 *vs* 5.97; Supplementary Table S6 and Supplementary Figure S4).

### Distribution of ATAC-Seq peaks within genomic features

**Figure 4:**
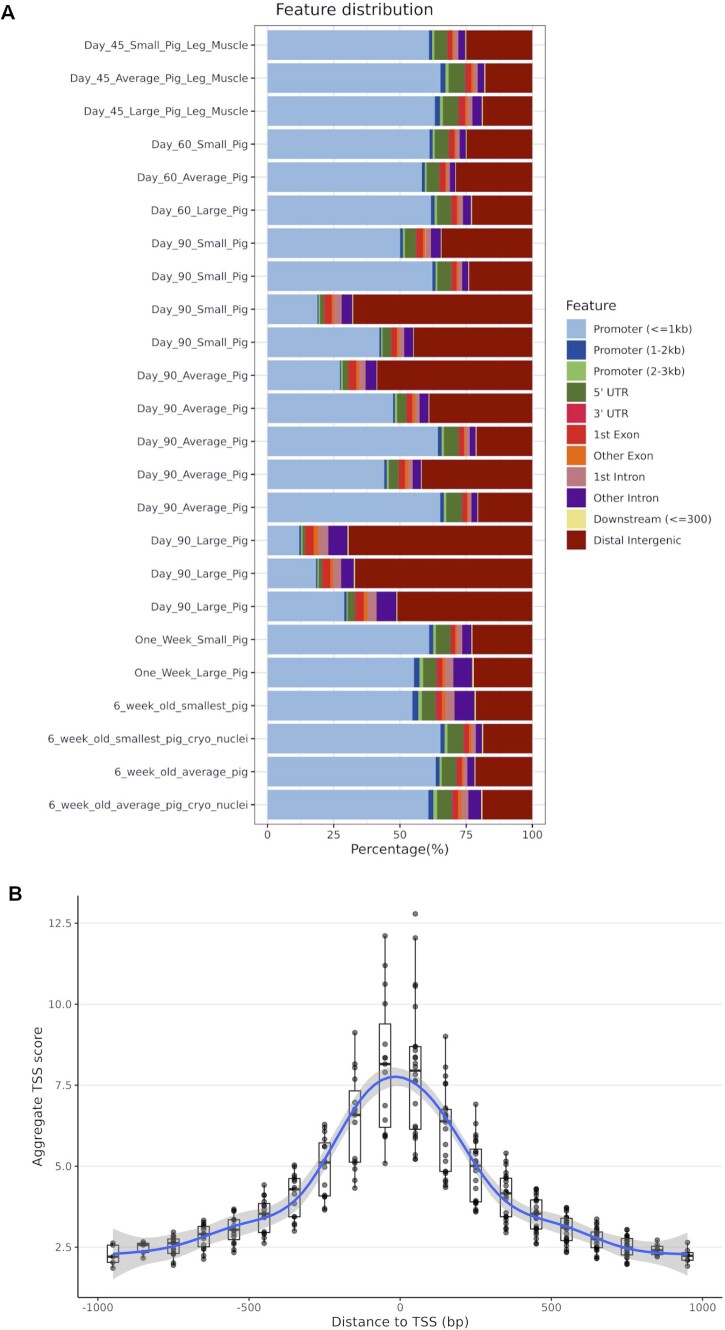
(A) Percentage of ATAC-Seq peaks within genomic features. The samples are sorted by the developmental timeline (day 45 to 6 weeks old from top to bottom). (B) A boxplot graph of TSS enrichment score of ATAC-Seq peaks and their relative distance from the TSS for all 24 samples. TSS score = the depth of TSS (each 100-bp window within 1000-bp flaking TSS)/the depth of flank ends (100 bp each end). TSS-E score for each library = max [mean (TSS score in each window)] calculated by ATAC-SeqQC v1.14.4 (Ou *et al.* 2018).

The feature distribution of the ATAC-Seq peaks in all 24 samples is shown in Figure 4A. On average >6500 peaks (including overlapping regions) were called in each sample (min: 2.34e+03, max: 1.42e+04, median: 6.38e+03, mean ± SD: 6.72e+03 ± 3.63e+03). More than 52% of the peaks were located in promoter regions in the majority of samples (19/24). There was a slight negative trend between increase in library size (depth of sequencing) and in the number of the peaks called (linear regression: slope = −6e−05 and *R*^2^ = 0.22). Detailed metrics are found in Supplementary File S2. In five samples [day 90: large (*n* = 3), day 90: average (*n* = 1), and day 90: small (*n* = 1)] most peaks were in distal intergenic regions (Figure 4A). We could not find any batch effect, in nuclei extraction or library preparation that might account for this and as such concluded that this variation was related to the samples themselves. There was also little observable difference in how the ATAC-Seq peaks were distributed within the genome of the libraries from cryopreserved nuclei relative to the libraries from flash-frozen tissue (Figure 4A). The breakdown of genomic feature categories in which peaks were located is presented in Table 2.

**Table 2:** The frequency of ATAC-Seq peaks in each genomic feature category annotated by ChIPseeker and averaged across samples (3766 annotated peaks from a total of 4661 peaks)

Features	Frequency (% mean ± SD)
Promoter (1–2 kb)	1.08 ± 0.52
Promoter (≤1 kb)	50.05 ± 16.96
Promoter (2–3 kb)	0.74 ± 0.28
5′ UTR	4.27 ± 1.64
3′ UTR	0.45 ± 0.25
1st exon	2.07 ± 0.59
Other exon	1.08 ± 0.32
1st Intron	3.77 ± 0.78
Downstream (≤3 kb)	0.23 ± 0.08
Distal intergenic	34.33 ± 17.29

### Proximity of ATAC-Seq peaks to transcription starts sites

The transcription start site enrichment score (TSS-ES) was used to validate the presence of the ATAC-Seq signal flanking transcription starts site (TSS) (Figure 4B). ATAC-Seq libraries had a TSS-ES of 8.03 ± 2.14 (mean ± SD) on average (min 5.21; max 12.8). As noted above, the ATAC-Seq TSS-ES was higher for libraries prepared from cryopreserved nuclei relative to flash-frozen tissue. All libraries showed a uniform distribution of the TSS scores flanking TSSs as shown in Figure 4B.

### Differential peak analysis of ATAC-Seq read counts using a consensus set of peaks

Differential peak analysis revealed 377 ATAC-Seq peaks from the consensus peak set in which the read count differed significantly between the developmental time points. These peaks were annotated using *Sscrofa11.1* corresponding to 724 unique transcripts (245 unique genes). One hundred and nine peaks were in unannotated intergenic regions. Nearly half of the peaks exhibiting differential read counts, between developmental time points, were in intronic regions and only 11.1% resided in promoters as shown in Table 3.

**Table 3:** Genomic feature distribution of ATAC-Seq peaks where read counts were significantly different between the day 45, day 60, day 90, 1-week, and 6-week time points

Features	Count	Frequency (%)
Intronic	185	49.1
Intergenic	109	28.9
Promoter	42	11.1
3′ UTR	12	3.18
Proximal	10	2.65
5′ UTR	8	2.12
CDS	8	2.12
Exonic	3	0.79

**Figure 5:**
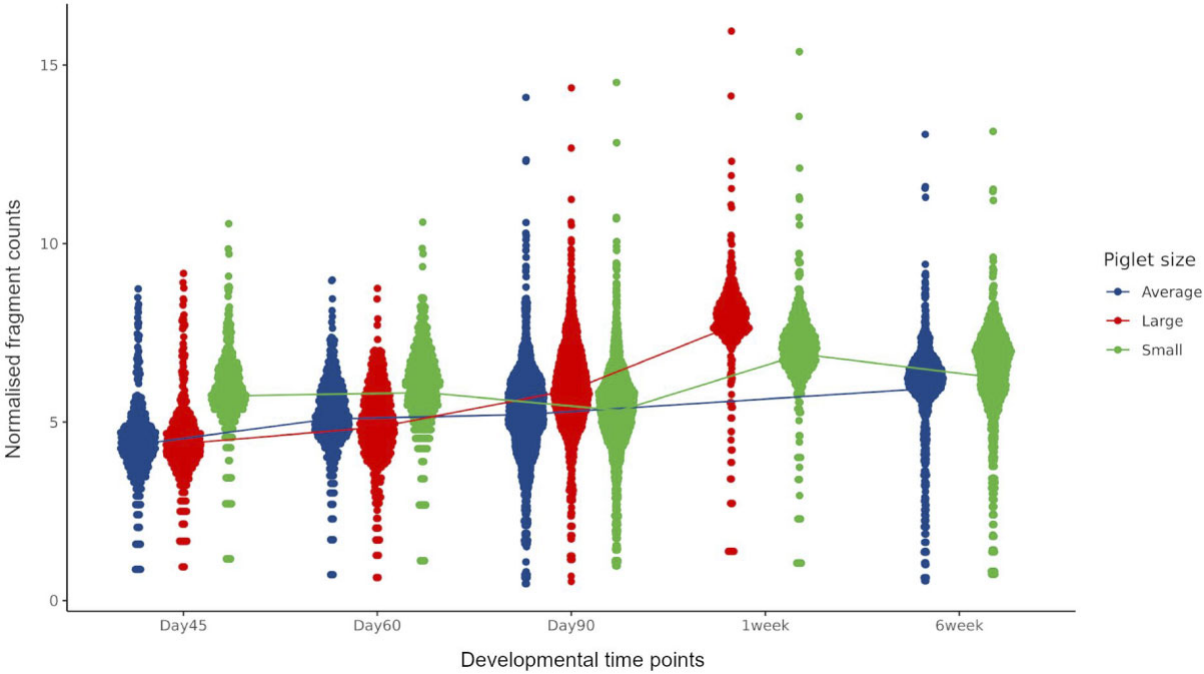
Normalized fragment counts plotted across developmental time points. Differential peak analysis of ATAC-Seq peaks across time points, including piglet size and litter as fixed variables in the DESeq2 LRT model. Significantly differentially expressed peaks (DEPs; *n* = 377 corresponding to 245 genes; FDR <0.1 and absolute log2FC >2) are plotted for each time point and colored by piglet size. The line represents the average normalized read counts per time point for all DEPs.

The read counts for the peaks that differed significantly between time points are shown in Figure 5 as normalized fragment counts. A detailed list of these peaks is included in Supplementary File S3. To test whether the fragment count distribution between the piglet sizes was different (Figure 5), we used ANOVA and found no significant differences between any of the pairwise comparisons (ANOVA + Tukey HSD, *P*-value >0.05).

### TF activity footprinting of the ATAC-Seq peaks (time and piglet size)

**Figure 6:**
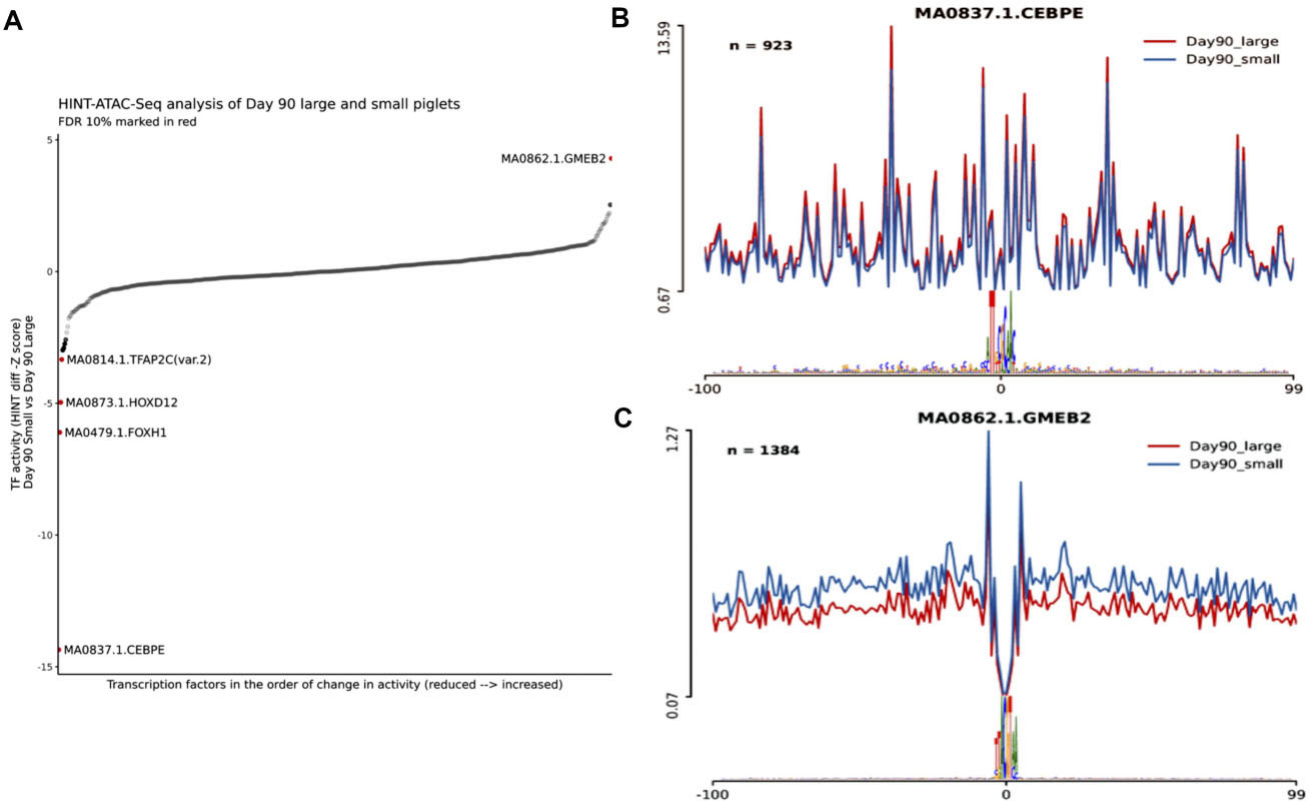
HINT pipeline analysis of the ATAC-Seq dataset for day 90 samples compared between large and small piglet size. (A) Differential TF activity between two piglet sizes at day 90 sorted by the HINT *z*-score value. The red dots are statistically significant (FDR 10%) showing hyperactivity of *GMEB2* in the small size piglets muscle tissues along with lowered activity of *TFAP2C*, *HOXD12*, *FOXH1*, and *CEBPE* TFs (higher in the large size piglets). TF activity in the vicinity of the corresponding motif between large and small size piglets is shown in (B) for *CEBPE* and (C) for *GMEB2*.

TF footprinting analysis across the developmental time points did not show any significantly different HINT scores (Figure 6A). In the comparison between large and small piglet size at day 90 samples, five differentially active TFs (*GMEB2*, *TFAP2C(var.2)*, *HOXD12*, *FOXH1*, and *CEBPE*) were detected using JASPAR2020 database annotation. The TF *CEBPE* CCAAT-Enhancer-Binding Protein-Beta showed the most extreme HINT *z* score (HINT *z* score −14.35; Figure 6B). *CEBPE* is known to be upregulated after muscle injury and be highly associated with muscle strength in human and mouse models (Harries *et al.* 2012). However, the lack of visual evidence of a TF footprint in either small or large piglets (Figure 6B) indicates that the extreme HINT *z* score might be the result of a technical artifact. In comparison, *GMEB2*, a glucocorticoid receptor expression regulator (Kaul *et al.* 2000), was the only TF with significantly higher enrichment in the small size piglets (HINT *z* score 4.29) in comparison to the large piglets and showed visual evidence of a TF footprint (Figure 6C). Details of the TF footprinting are shown in Table 4.

**Table 4:** TF foot printing analysis of the ATAC-Seq dataset using HINT and the JASPAR annotation database

Motif	TF_activity	*Z*_score	*P*-values	FDR
MA0837.1.CEBPE	−0.71	−14.35	1.01E−46	5.59E−44
MA0479.1.FOXH1	−0.30	−6.10	1.04E−09	2.87E−07
MA0873.1.HOXD12	−0.24	−4.96	6.95E−07	1.28E−04
MA0862.1.GMEB2	0.21	4.29	1.74E−05	2.4E−03
MA0814.1.TFAP2C(var.2)	−0.16	−3.33	8.49E−04	9.3E−02

FDR, false discovery rate (10% was considered significant). Comparison was performed in day 90 samples small to large (S/L direction of activity value).

### Analysis of gene expression using RNA-Seq

**Figure 7:**
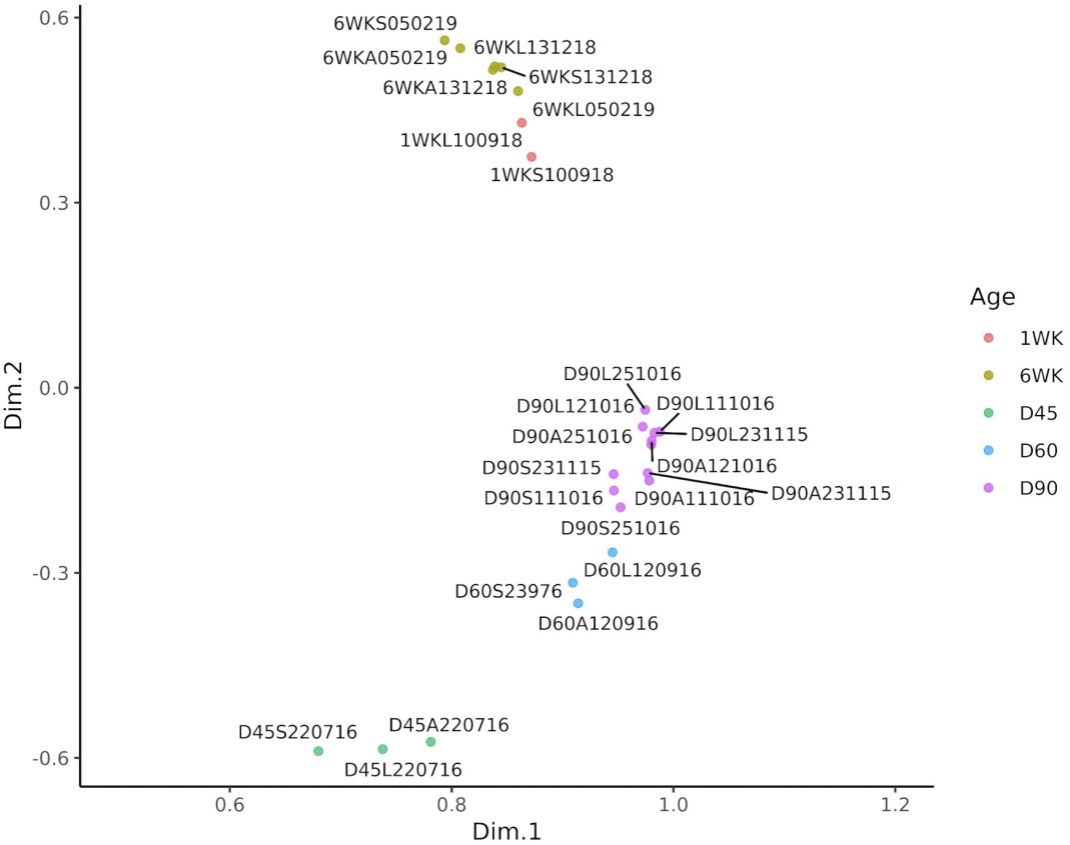
PCA of gene expression estimates (as TPM) from the RNA-Seq data for each of 25 samples. The samples cluster according to the developmental stage with clear separation of prenatal and postnatal samples. Prenatal: D45 = gestational day 45; D60 = gestational day 60; D90 = gestational day 90; postnatal: 1WK = neonatal 1 week old; 6WK = juvenile 6 weeks old.

We generated RNA-Seq data from the same muscle tissue samples that were used to generate the ATAC-Seq libraries, to link regions of open chromatin with gene expression. The transcript expression estimates for the muscle tissue samples from the five developmental time points (26 samples in total) were calculated as TPM reads using Kallisto. The TPM expression estimates were then investigated using PCA (Figure 7) to identify any samples that did not group as expected according to the developmental time point. The samples from each developmental time point clustered together as expected in the first two dimensions of the PCA (Figure 7).

### Analysis of genes that were differentially expressed between the three sizes of fetal piglet at day 90 of gestation

**Figure 8:**
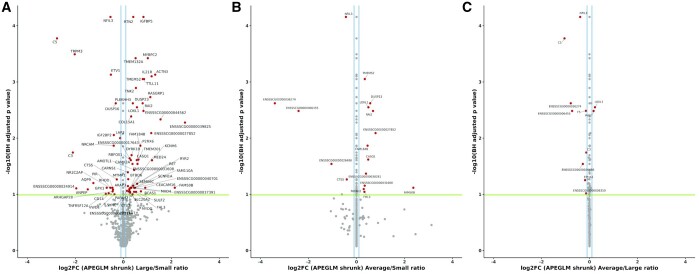
Differentially expressed genes (RNA-Seq) between large-, average-, and small-sized piglets at Day 90 of gestation. (A) Large *vs* small, (B) average *vs* small, and (C) average *vs* large. Differentially expressed genes are shown in red, the log2FC >0.1 in blue, and the significance threshold as a green line. The ApeGLM shrinkage method was used to normalized the log fold change plotted on the *x*-axis as described in Love *et al.* (2014).

Differential gene expression analysis was performed using the TPM values for the three sizes of fetal piglet (small, average, and large) at day 90 of gestation. Between the three sizes of fetal piglet 89 genes (FDR 10%) were found to be differentially expressed. When average- *vs* small-sized fetal piglets were compared, 58 upregulated and 31 downregulated genes were detected. When large- *vs* small-sized fetal piglets were compared, 54 upregulated and 35 downregulated genes were detected. Differentially expressed genes with an adjusted *P*-value (FDR <0.1) and log2FC ≥ 0.1 are annotated in Figure 8. The comparison between large- and small-sized fetal piglets resulted in the largest number (*n* = 89) of differentially expressed genes. The list of differentially expressed genes and detailed analysis metrics are found in Supplementary Table S5. Many of the genes that were differentially expressed between large and small, and average and small, fetal piglets are involved in skeletal muscle function and growth (Figure 8). The gene calsequestrin 1 (*CASQ1*), for example, which was 1.54-fold upregulated (log2FC 0.63 ± 0.17 adjusted *P*-value = 2.0e−02) in large-sized relative to small-sized fetal piglets is the skeletal muscle-specific member of the calsequestrin protein family and is highly expressed in skeletal muscle in adult pigs, see (http://biogps.org/pigatlas/; Freeman *et al.* 2012; Summers *et al.* 2020; BioGPS 2021). *MYBPC2*, a gene that encodes myosin-binding protein C, was also two-fold upregulated (log2FC 1.02 ± 0.22 adjusted *P*-value = 3.78e−04) in large-sized relative to small-sized fetal piglets (Figure 8). It has also been shown to be highly expressed in the muscle of pigs (see http://biogps.org/pigatlas/; Freeman *et al.* 2012; Summers *et al.* 2020; BioGPS 2021). The muscle-specific TF myogenin (*MYOG*) was downregulated (log2FC 0.28 ± 0.09 adjusted *P*-value = 9.0e−02), in small-sized relative to large-sized fetal piglets (Figure 8).

### Overlay of the RNA-Seq differentially expressed genes and ATAC-Seq peaks from large *vs* small fetal piglets at day 90 of gestation

**Figure 9:**
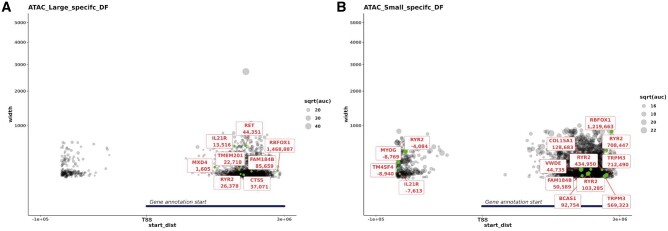
Proximity of ATAC-Seq peaks specific to large (A) and small (B) piglets and differentially expressed genes. Differentially expressed genes are marked in green. The *x*-axis (start_dist) is the distance from the start of the gene model to the start of the ATAC-Seq peak, for +ve values the peak is either within the gene or within 10 kb of the 3′ end, and for −ve values the peak is within 10 kb of the 5′ end of the gene. The *y*-axis indicates the width of the peak. As the *y*-axis represents the width of the peak, the larger the node the wider the ATAC-Seq peak.

A further overlay of the ATAC-Seq and RNA-Seq datasets was performed for the day 90 large- and small-sized fetal piglets. ATAC-Seq peaks annotated using the Sscrofa11.1 Ensembl gene track information (black) and differentially expressed genes between the large- *vs* small-sized fetal piglets at day 90 (green) are shown in Figure 9. This analysis allowed us to determine which of the differentially expressed genes had an ATAC-Seq peak that was specific to either large- or small-sized piglets in its vicinity. The distribution of ATAC-Seq peaks around TSSs (within a 3-kb distance) was plotted for peaks specific to the large-sized fetal piglets (Figure 9A), or specific to the small-sized fetal piglets (Figure 9B). Size-specific peaks within the 5′UTR of four differentially expressed genes, *MYOG*, ryanodine receptor 2 (*RYR2*), transmembrane 4L six family member 4 (*TM4SF4*), and interleukin 21 receptor (*IL21R*; Figure 8), were only observed in the small fetal piglets (Figure 9B). There was no evidence of size-specific peaks near these genes in the large-sized fetal piglets (Figure 9B). Of the four genes, *MYOG* is known to be highly expressed in skeletal muscle tissue (see http://biogps.org/pigatlas/; Freeman *et al.* 2012; Summers *et al.* 2020; BioGPS 2021). In some cases, a size-specific ATAC-Seq peak was located within the 5′ UTR of a gene that was involved in muscle growth and downregulated in small relative to large piglets. *MYOG*, for example, was downregulated in small-sized fetal piglets (Figure 8), with a regulatory region 315 bp in size 8769 bp upstream of the TSS, that was present in the small-sized piglets (Figure 9A) but absent in the large-sized fetal piglets (Figure 9B).

## Discussion

In this study, we used ATAC-Seq and RNA-Seq to improve our understanding of gene expression and regulation in developing pig muscle. We generated open chromatin profiles, in the form of ATAC-Seq peaks, for semitendinosus muscle from piglets from five developmental stages and compared these with gene expression profiles from the same tissue samples.

Of the 4661 ATAC-Seq peaks representing regions of open chromatin, that were identified in this study, >50% were within 1 kb of known transcription start sites. This result is consistent with studies across different species (Foissac *et al.* 2019; Yue *et al.* 2021). A study of *longissimus dorsi* muscle from pig embryos at days 45, 70, and 100 conducted by Yue *et al.* (2021) showed that 30%, 21%, and 14% of the peaks were identified in promoter regions, respectively. Of these peaks, 91% mapped to within −1 kb and +100 bp of the TSS (Yue *et al.* 2021). A cross-species analysis of ATAC-Seq data showed that in mice, goats, cattle, pigs, and chicken, 10–15% of ATAC-Seq peaks were located within up to 5 kb of the TSS and were therefore considered as promoters (Foissac *et al.* 2019). The results from our study showed that the majority of the ATAC-Seq peak frequency was located within ±1 kb of the TSS, with the remaining peaks located primarily within distal intergenic regions, particularly at day 90 of gestation. The distribution of ATAC-Seq peaks in intergenic regions at day 90 in large- and small-sized piglets indicated that piglets of different sizes show changes in genome regulation primarily at intergenic sites, which could be indicative of differential enhancer activity. Day 90 is a critical stage of muscle development when fiber formation ceases and muscle growth accelerates through fiber hypertrophy (Oksbjerg *et al.* 2004). Significant upregulation of genes involved in muscle growth also occurs at day 90 (Zhao *et al.* 2015; Ayuso *et al.* 2016; Yue *et al.* 2021). As such chromatin may be more open at this developmental stage to allow TF binding prior to the rapid muscle growth that occurs during the early postnatal period (Rudar *et al.* 2019).

Developmental stage-specific patterns in chromatin accessibility were observed in this study. Differential read count analysis revealed 377 ATAC-Seq defined genomic regions where chromatin accessibility differed significantly across developmental time points. Other similar studies (*e.g.*Yue *et al.* 2021) have reported widespread increases in accessible chromatin and increasing regulatory complexity in developing pig embryos through days 45, 75, and 100 of gestation. Studies profiling open chromatin in preimplantation embryos found global differences in chromatin accessibility between embryo stages in humans (Wu *et al.* 2018; Liu *et al.* 2019) and cattle (Halstead *et al.* 2020c). ATAC-Seq datasets for postimplantation embryos in humans and other mammalian species are limited. ChIP-Seq analyses of a wide variety of histone markers in the brain, heart, and liver of early human embryos identified developmental stage-specific patterns in the epigenome (Yan *et al.* 2016). The sample size in our study was small, with only a few biological replicates for most developmental time points, except for day 90. Even so our results are in agreement with other recent studies (*e.g.*, Yue *et al.* 2021) and indicate that chromatin accessibility and regulation of gene expression change throughout development in pig semitendinosus muscle.

Analyzing tissue samples from several prenatal time points can help to determine when during early development tissue-specific gene regulation might have an effect on phenotype. For example, in this study, TF footprint analysis showed that the TF *GMEB2*, which increases sensitivity to glucocorticoids (Kaul *et al.* 2000), had significantly higher TF activity in small-sized relative to large-sized piglets at day 90 of gestation. This finding is potentially phenotypically relevant because low birth weight piglets have been shown to have higher *in utero*-cortisol levels than their normal birth weight litter mates (Roelofs *et al.* 2019). Given the small sample size of this study, this result should be treated with some caution, but it does indicate that further investigation of day 90 of gestation with a larger and more balanced experiment would be warranted.

In our study, we also compared gene expression and chromatin openness between fetal piglets of different sizes (small, average, and large) at day 90 of gestation. Other studies have used a similar approach to investigate the effect of histone modification on the expression of genes involved in placental development in pigs (Han *et al.* 2019) and chromatin accessibility in prenatal muscle development (Yue *et al.* 2021). Differences in open chromatin were reflected in the expression of genes involved in muscle growth. Analysis of the RNA-Seq data revealed that genes associated with muscle growth, including *CASQ1*, *MYBPC2*, and *MYOG*, were differentially expressed in large relative to small piglets. Differential expression of myogenic genes (*e.g.*, *MYOG*) in pig muscle has been previously reported by Felicioni *et al.* (2020) who compared intrauterine growth-restricted piglets and normal weight piglets. *CASQ1* encodes the skeletal muscle-specific member of the calsequestrin protein family, is related to muscle metabolism, and has been shown to be highly expressed in fat pig breeds (Zhao *et al.* 2011). In this study, *CASQ1* was upregulated in large-sized relative to small-sized fetal piglets. *MYBPC2* encodes the fast isoform of the myosin-binding protein C family (Weber *et al.* 1993). In Piedmontese (*GDF8* mutant) cattle, *MYBPC2* is highly expressed in fetal muscle, reflecting fast glycolytic fiber structural differentiation (Lehnert *et al.* 2007). In this study, *MYBPC2* was highly upregulated in large *vs* small sized fetal piglets, potentially reflecting a greater proportion of fast glycolytic muscle fibers.

*MYOG* is essential for myoblast fusion during muscle development (UniProt 2021) and associated with QTLs, for body weight at birth (AnimalQTLdb 2021a) and backfat thickness (AnimalQTLdb 2021b), according to Genome Wide Association Studies (Xue and Zhou 2006). *MYOG* was downregulated in small-sized fetal piglets relative to large-sized fetal piglets. Other studies measuring gene expression also found that *MYOG* was downregulated in muscle cell types from low birth weight piglets relative to their “normal”-sized litter mates (Felicioni *et al.* 2020; Stange *et al.* 2020) and in pigs with high levels of intramuscular fat relative to those with average levels (Lim *et al.* 2017). When we compared the ATAC-Seq and RNA-Seq data, we identified an ATAC-Seq peak within the 5′ UTR of *MYOG* (315 bp in size, 8769 bp upstream of the TSS) that was present in the small-sized fetal piglets but missing from the large-sized fetal piglets for day 90 of gestation. This result was surprising because *MYOG* was downregulated in the small-sized relative to the large-sized fetal piglets and as such we would have expected the chromatin to be less accessible and no peak to be present in the small-sized piglets. In future work, we plan to remove this peak using CRISPR genome editing and measure the effect on muscle progenitor cells in culture.

The datasets we have generated for this study provide important functional annotation information, providing novel annotation tracks for regions of open chromatin in the pig reference genome (Sscrofa 11.1). We have made the datasets available in the public archives and via the FAANG Data Portal for this purpose. The datasets also provide a foundation for incorporating functional information in statistical analyses, to increase the precision and power with which we can fine map high-quality causal variants in pigs. This would make it possible to increase the accuracy of genomic selection and the efficiency with which breeding turns genetic variation into genetic gain. Further investigation of genetic variants within the regulatory regions identified in this study would help to validate whether they might be driving muscle and growth phenotypes. Recently, a functional regulatory variant was identified in *MYH3* that influences muscle fiber type composition and intramuscular fat content in pigs (Cho *et al.* 2019). The next stage of the study is to leverage the ATAC-Seq and RNA-Seq data with a very large dataset of genetic variants from production pigs to determine whether any trait-linked variants are located within the open chromatin regions we have identified for muscle tissue. The characterization of regulatory and expressed regions of the genome in muscle tissues also provides a basis for genome editing to promote functional genomic variants in pig breeding programs (Jenko *et al.* 2015; Hickey *et al.* 2016; Johnsson *et al.* 2019), providing a route to application for FAANG data.

## Conclusions

The dataset that we have generated provides a powerful foundation to investigate how the genome is regulated in production pigs and contributes valuable functional annotation information, for global FAANG efforts, and to define and predict the effects of genetic variants in pig breeding programs. The outcomes of the study will: (1) help us to understand the molecular drivers of muscle growth in pigs; (2) provide a foundation for functionally validating target genomic regions in in vitro systems; and (3) identify high-quality causative variants for muscle and growth traits with the goal of harnessing genetic variation and turning it into sustainable genetic gain in pig breeding programs.

## Data availability

The raw sequence data for the ATAC-Seq samples (*n* = 24) are available via the European Nucleotide Archive (ENA) under accession number PRJEB41485. Details of all samples processed for the RNA-Seq dataset (*n* = 26) can be accessed via the ENA under accession number PRJEB41488. The sample metadata is available via the BioSamples database under sample accession numbers SAMEA7178119, SAMEA7178120, SAMEA7178122, SAMEA7178123, SAMEA7178124, SAMEA7178125, SAMEA7178126, SAMEA7178127, SAMEA7178134, SAMEA7178138, SAMEA7178142, SAMEA7178149, SAMEA7178150, SAMEA7178153, SAMEA7178159, SAMEA7178160, SAMEA7178164, SAMEA7178178, SAMEA7178179, SAMEA7178180, SAMEA7178182, SAMEA7178183, SAMEA7178184, SAMEA7178185, SAMEA7178187, and SAMEA7178188. These datasets are curated and submitted to FAANG data portal according to FAANG’s sample and experimental guidelines (Harrison *et al.* 2018). All the sample, experiment, and analysis protocols for this study are also available through the FAANG Data Coordination Centre via the following: FAANG-SOP1 (2021), FAANG-SOP2 (2021), FAANG-SOP3 (2021), FAANG-SOP4 (2021), FAANG-SOP5 (2021), FAANG-SOP6 (2021), FAANG-SOP7 (2021), and FAANG-SOP8 (2021). All of the supplementary tables, figures, and files are also available from https://doi.org/10.6084/m9.figshare.13562285. The bioinformatic pipelines used for processing the ATAC-Seq (mapping and peak calling) and RNA-Seq (transcript-level expression analysis) are available via a code repository in Salavati (2021).

Supplementary material is available at *G3* online.

## Supplementary Material

jkab424_Supplemental_Figure_S1

jkab424_Supplemental_Figure_S2

jkab424_Supplemental_Figure_S3

jkab424_Supplemental_Figure_S4

jkab424_Supplemental_File_S1

jkab424_Supplemental_File_S2

jkab424_Supplemental_File_S3

jkab424_Supplemental_Material_Legends

jkab424_Supplemental_Table_1

jkab424_Supplemental_Table_2

jkab424_Supplemental_Table_3

jkab424_Supplemental_Table_4

jkab424_Supplemental_Table_5

jkab424_Supplemental_Table_6

## References

[jkab424-B1] Aiello D , Patel K, Lasagna E. 2018. The myostatin gene: an overview of mechanisms of action and its relevance to livestock animals. Anim Genet. 49:505–519. doi:10.1111/age.12696. 30125951

[jkab424-B2] Alexandre PA , Naval-Sánchez M, Menzies M, Nguyen LT, Porto-Neto LR, et al 2021. Chromatin accessibility and regulatory vocabulary across indicine cattle tissues. Genome Biol. 22:273. doi:10.1186/s13059-021-02489-7. 34548076 PMC8454054

[jkab424-B3] Anders S , Pyl PT, Huber W. 2015. HTSeq—a Python framework to work with high-throughput sequencing data. Bioinformatics. 31:166–169. doi:10.1093//btu638. 25260700 PMC4287950

[jkab424-B4] Andersson L , Archibald AL, Bottema CD, Brauning R, Burgess SC, et al; FAANG Consortium. 2015. Coordinated international action to accelerate genome-to-phenome with FAANG, the Functional Annotation of Animal Genomes project. Genome Biol. 16:57. doi:10.1186/s13059-015-0622-4. 25854118 PMC4373242

[jkab424-B5] Andrews S. 2010. FastQC: A Quality Control Tool for High Throughput Sequence Data. http://www.bioinformatics.babraham.ac.uk/projects/fastqc/.

[jkab424-B6] AnimalQTLdb. 2021a. Pig QTL Database # 8656. (Accessed: 2021 October 29). https://www.animalgenome.org/cgi-bin/QTLdb/SS/qdetails?QTL_ID=8656.

[jkab424-B7] AnimalQTLdb. 2021b. Pig QTL Database # 8657. (Accessed: 2021 October 29). https://www.animalgenome.org/cgi-bin/QTLdb/SS/qdetails?QTL_ID=8657.

[jkab424-B8] Ashmore CR , Addis PB, Doerr L. 1973. Development of muscle fibers in the fetal pig. J Anim Sci. 36:1088–1093. doi:10.2527/jas1973.3661088x. 4268264

[jkab424-B9] Ayuso M , Fernández A, Núñez Y, Benítez R, Isabel B, et al 2016. Developmental stage, muscle and genetic type modify muscle transcriptome in pigs: effects on gene expression and regulatory factors involved in growth and metabolism. PLoS One. 11:e0167858. doi:10.1371/journal.pone.0167858. 27936208 PMC5148031

[jkab424-B10] Benjamini Y , Hochberg Y. 1995. Controlling the false discovery rate: a practical and powerful approach to multiple testing on JSTOR. Source J R Stat Soc Ser B. 57:289–300.

[jkab424-B11] BioGPS. 2021. BioGPS—Your Gene Portal System. BioGPS Blog. (Accessed: 2021 October 29). http://biogps.org/pigatlas/.

[jkab424-B12] Bolger AM , Lohse M, Usadel B. 2014. Trimmomatic: a flexible trimmer for Illumina sequence data. Bioinformatics. 30:2114–2120. doi:10.1093/bioinformatics/btu170. 24695404 PMC4103590

[jkab424-B13] Bray NL , Pimentel H, Melsted P, Pachter L. 2016. Near-optimal probabilistic RNA-seq quantification. Nat Biotechnol. 34:525–527. doi:10.1038/nbt.3519. 27043002

[jkab424-B14] Broad Institute. 2019. Picard Toolkit. Broad Institute, GitHub Repos. Cambridge, Massachusetts: Broad Institute. https://broadinstitute.github.io/picard/ (Accessed: 2021 December 17).

[jkab424-B15] Buenrostro JD , Giresi PG, Zaba LC, Chang HY, Greenleaf WJ. 2013. Transposition of native chromatin for fast and sensitive epigenomic profiling of open chromatin, DNA-binding proteins and nucleosome position. Nat Methods. 10:1213–1218. 24097267 10.1038/nmeth.2688 PMC3959825

[jkab424-B16] Buenrostro JD , Wu B, Chang HY, Greenleaf WJ. 2015. ATAC-seq: a method for assaying chromatin accessibility genome-wide. Curr Protoc Mol Biol. 109:21.29.1–21.29.9. doi:10.1002/0471142727.mb2129s109. PMC4374986 25559105

[jkab424-B17] Cho I-C , Park H-B, Ahn JS, Han S-H, Lee J-B, et al 2019. A functional regulatory variant of *MYH3* influences muscle fiber-type composition and intramuscular fat content in pigs. PLOS Genet. 15:e1008279. 31603892 10.1371/journal.pgen.1008279 PMC6788688

[jkab424-B18] Clark EL , Archibald AL, Daetwyler HD, Groenen MAM, Harrison PW, et al 2020. From FAANG to fork: application of highly annotated genomes to improve farmed animal production. Genome Biol. 21:285. doi:10.1186/s13059-020-02197-8. 33234160 PMC7686664

[jkab424-B19] Corces MR , Trevino AE, Hamilton EG, Greenside PG, Sinnott-Armstrong NA, et al 2017. An improved ATAC-seq protocol reduces background and enables interrogation of frozen tissues. Nat Methods. 14:959–962. doi:10.1038/nmeth.4396. 28846090 PMC5623106

[jkab424-B20] Davie K , Jacobs J, Atkins M, Potier D, Christiaens V, et al 2015. Discovery of transcription factors and regulatory regions driving in vivo tumor development by ATAC-seq and FAIRE-seq open chromatin profiling. PLoS Genet. 11:e1004994. doi:10.1371/journal.pgen.1004994. 25679813 PMC4334524

[jkab424-B21] Davis CA , Hitz BC, Sloan CA, Chan ET, Davidson JM, et al 2018. The encyclopedia of DNA elements (ENCODE): data portal update. Nucleic Acids Res. 46: D794–D801. doi:10.1093/nar/gkx1081. 29126249 PMC5753278

[jkab424-B22] Edinburgh U of 2020. Edinburgh Compute and Data Facility. (Accessed: 2020 July 6). https://www.ed.ac.uk/is/research-computing-service.

[jkab424-B23] Estany J , Ros-Freixedes R, Tor M, Pena RN. 2017. Triennial Growth and Development Symposium: genetics and breeding for intramuscular fat and oleic acid content in pigs. J Anim Sci. 95:2261–2271. doi:10.2527/jas.2016.1108. 28727022

[jkab424-B24] Ewels P , Magnusson M, Lundin S, Käller M. 2016. MultiQC: summarize analysis results for multiple tools and samples in a single report. Bioinformatics. 32:3047–3048. doi:10.1093/bioinformatics/btw354. 27312411 PMC5039924

[jkab424-B33] FAANG. 2021. FAANG Data Portal. (Accessed: 2021 October 29). https://data.faang.org/.

[jkab424-B25] FAANG-SOP1. 2021. Collection of tissue samples for ATAC-Seq and RNA-Seq from large animals. (Accessed: 2021 October 29). https://data.faang.org/api/fire_api/samples/ROSLIN_SOP_Collection_of_tissue_samples_for_ATAC-Seq_and_RNA-Seq_from_large_animals_20200618.pdf.

[jkab424-B26] FAANG-SOP2. 2021. Cryo-preservation of nuclei from tissue for ATAC-Seq using the GentleMACS system (adapted from (Halstead et al., 2020)). (Accessed: 2021 October 29). https://data.faang.org/api/fire_api/samples/ROSLIN_SOP_Cryopreservation_of_Nuclei_for_ATACSeq_using_GentleMACS_20201119.pdf.

[jkab424-B27] FAANG-SOP3. 2021. ATAC-Seq Isolation of Nuclear DNA and Tagmentation from Cryo Preserved Nuclei from Pig Muscle Cells. (Accessed: 2021 October 29). https://data.faang.org/api/fire_api/samples/ROSLIN_SOP_ATAC-Seq_DNAIsolationandTagmentation_Cryopreserved_Muscle_Nuclei_Preparations_20200720.pdf.

[jkab424-B28] FAANG-SOP4. 2021. ATAC-Seq Isolation of Nuclear DNA from frozen tissue and tagmentation. (Accessed: 2021 October 29). https://data.faang.org/api/fire_api/samples/ROSLIN_SOP_ATAC_Seq_DNAIsolationandTagmentation_Frozen_Muscle_Tissue_20200720.pdf.

[jkab424-B29] FAANG-SOP5. 2021. ATAC-Seq Library Preparation and Size Selection. (Accessed: 2021 October 29). https://data.faang.org/api/fire_api/samples/ROSLIN_SOP_ATAC-Seq_LibraryPreparationandSizeSelection_20200720.pdf.

[jkab424-B30] FAANG-SOP6. 2021. Isolation of total RNA from frozen tissue samples. (Accessed: 2021 October 29). https://data.faang.org/api/fire_api/samples/ROSLIN_SOP_RNA_IsolationoftotalRNA fromfrozentissuesamples_20200720.pdf.

[jkab424-B31] FAANG-SOP7. 2021. Assay for Transposase-Accessible Chromatin (ATAC-Seq) data processing workflow. (Accessed: 2021 October 29). https://data.faang.org/api/fire_api/analyses/ROSLIN_SOP_ATAC-Seq_analysis_pipeline_20201113.pdf.

[jkab424-B32] FAANG-SOP8. 2021. mRNA-Seq data processing workflow. (Accessed: 2021 October 29). https://data.faang.org/api/fire_api/analyses/ROSLIN_SOP_RNA-Seq_analysis_pipeline_20201113.pdf.

[jkab424-B34] Felicioni F , Pereira AD, Caldeira-Brant AL, Santos TG, Paula TMDD, Magnabosco D, et al 2020. Postnatal development of skeletal muscle in pigs with intrauterine growth restriction: morphofunctional phenotype and molecular mechanisms. J Anat. 236:840–853. doi:10.1111/joa.13152. 31997379 PMC7163581

[jkab424-B35] Foissac S , Djebali S, Munyard K, Vialaneix N, Rau A, et al 2019. Multi-species annotation of transcriptome and chromatin structure in domesticated animals. BMC Biol. 17:108. doi:10.1186/s12915-019-0726-5. 31884969 PMC6936065

[jkab424-B36] Fornes O , Castro-Mondragon JA, Khan A, Van Der Lee R, Zhang X, et al 2020. JASPAR 2020: update of the open-access database of transcription factor binding profiles. Nucleic Acids Res. 48: D87–D92. doi:10.1093/nar/gkz1001. 31701148 PMC7145627

[jkab424-B37] Freeman TC , Ivens A, Baillie JK, Beraldi D, Barnett MW, et al 2012. A gene expression atlas of the domestic pig. BMC Biol. 10:90. doi:10.1186/1741-7007-10-90. 23153189 PMC3814290

[jkab424-B38] Giuffra E , Tuggle CK, FAANG Consortium. 2019. Functional Annotation of Animal Genomes (FAANG): current achievements and roadmap. Annu Rev Anim Biosci. 7:65–88. doi:10.1146/annurev-animal-020518-114913. 30427726

[jkab424-B39] Halstead MM , Kern C, Saelao P, Chanthavixay G, Wang Y, et al 2020a. Systematic alteration of ATAC-seq for profiling open chromatin in cryopreserved nuclei preparations from livestock tissues. Sci Rep. 10:5230. doi:10.1038/s41598-020-61678-9. 32251359 PMC7089989

[jkab424-B40] Halstead MM , Kern C, Saelao P, Wang Y, Chanthavixay G, et al 2020b. A comparative analysis of chromatin accessibility in cattle, pig, and mouse tissues. BMC Genomics. 21:1–16. doi:10.1186/s12864-020-07078-9. PMC7541309 33028202

[jkab424-B41] Halstead MM , Ma X, Zhou C, Schultz RM, Ross PJ. 2020c. Chromatin remodeling in bovine embryos indicates species-specific regulation of genome activation. Nat Commun. 11:1–16. doi:10.1038/s41467-020-18508-3. 32943640 PMC7498599

[jkab424-B42] Han K , Ren R, Cao J, Zhao S, Yu M. 2019. Genome-wide identification of histone modifications involved in placental development in pigs. Front Genet. 10:277. 30984246 10.3389/fgene.2019.00277 PMC6449610

[jkab424-B43] Harries LW , Pilling LC, Hernandez LDG, Bradley-Smith R, Henley W, et al 2012. CCAAT-enhancer-binding protein-beta expression in vivo is associated with muscle strength. Aging Cell. 11:262–268. doi:10.1111/j.1474-9726.2011.00782.x. 22152057 PMC3486692

[jkab424-B44] Harrison PW , Fan J, Richardson D, Clarke L, Zerbino D, et al 2018. FAANG, establishing metadata standards, validation and best practices for the farmed and companion animal community. Anim Genet. 49:520–526. doi:10.1111/age.12736. 30311252 PMC6334167

[jkab424-B45] Hickey JM , Bruce C, Whitelaw A, Gorjanc G. 2016. Promotion of alleles by genome editing in livestock breeding programmes. J Anim Breed Genet. 133:83–84. doi:10.1111/jbg.12206. 26995217

[jkab424-B46] Jenko J , Gorjanc G, Cleveland MA, Varshney RK, Whitelaw CBA, et al 2015. Potential of promotion of alleles by genome editing to improve quantitative traits in livestock breeding programs. Genet Sel Evol. 47:55. doi:10.1186/s12711-015-0135-3. 26133579 PMC4487592

[jkab424-B47] Gaspar JM. 2020. Genrich: Detecting Sites of Genomic Enrichment. https://github.com/jsh58/Genrich.

[jkab424-B48] Johnsson M , Gaynor RC, Jenko J, Gorjanc G, de Koning D-J, et al 2019. Removal of alleles by genome editing (RAGE) against deleterious load. Genet Sel Evol. 51:14. doi:10.1186/s12711-019-0456-8. 30995904 PMC6472060

[jkab424-B49] Kaul S , Blackford JA, Chen J, Ogryzko VV, Simons SS. 2000. Properties of the glucocorticoid modulatory element binding proteins *GMEB-1* and *-2*: potential new modifiers of glucocorticoid receptor transactivation and members of the family of KDWK proteins. Mol Endocrinol. 14:1010–1027. doi:10.1210/mend.14.7.0494. 10894151

[jkab424-B50] Lehnert SA , Reverter A, Byrne KA, Wang Y, Nattrass GS, et al 2007. Gene expression studies of developing bovine longissimusmuscle from two different beef cattle breeds. BMC Dev Biol. 7:95. doi:10.1186/1471-213X-7-95. 17697390 PMC2031903

[jkab424-B51] Li H. 2011. A statistical framework for SNP calling, mutation discovery, association mapping and population genetical parameter estimation from sequencing data. Bioinformatics. 27:2987–2993. doi:10.1093/bioinformatics/btr509. 21903627 PMC3198575

[jkab424-B52] Li H , Handsaker B, Wysoker A, Fennell T, Ruan J, et al; 1000 Genome Project Data Processing Subgroup. 2009. The sequence alignment/map format and SAMtools. Bioinformatics. 25:2078–2079. doi:10.1093/bioinformatics/btp352. 19505943 PMC2723002

[jkab424-B53] Li Y , Li B, Yang M, Han H, Chen T, et al 2020. Genome-wide association study and fine mapping reveals candidate genes for birth weight of Yorkshire and Landrace pigs. Front Genet. 11:183. doi:10.3389/fgene.2020.00183. 32292414 PMC7118202

[jkab424-B54] Li Z , Schulz MH, Look T, Begemann M, Zenke M, et al 2019. Identification of transcription factor binding sites using ATAC-seq. Genome Biol. 20:1–21. doi:10.1186/s13059-019-1642-2. 30808370 PMC6391789

[jkab424-B55] Lim KS , Lee KT, Park JE, Chung WH, Jang GW, et al 2017. Identification of differentially expressed genes in longissimus muscle of pigs with high and low intramuscular fat content using RNA sequencing. Anim Genet. 48:166–174. doi:10.1111/age.12518. 27928823

[jkab424-B56] Liu L , Leng L, Liu C, Lu C, Yuan Y, et al 2019. An integrated chromatin accessibility and transcriptome landscape of human pre-implantation embryos. Nat Commun. 10:364. doi:10.1038/s41467-018-08244-0. 30664750 PMC6341076

[jkab424-B57] Love MI , Huber W, Anders S. 2014. Moderated estimation of fold change and dispersion for RNA-seq data with DESeq2. Genome Biol. 15:550. doi:10.1186/s13059-014-0550-8. 25516281 PMC4302049

[jkab424-B58] Luo X-J , Liu J, Luo Y, Zhang X-L, Wu J-P, et al 2009. Polybrominated diphenyl ethers (PBDEs) in free-range domestic fowl from an e-waste recycling site in South China: levels, profile and human dietary exposure. Environ Int. 35:253–258. doi:10.1016/j.envint.2008.06.007. 18676020

[jkab424-B59] Oksbjerg N , Gondret F, Vestergaard M. 2004. Basic principles of muscle development and growth in meat-producing mammals as affected by the insulin-like growth factor (*IGF*) system. Domest Anim Endocrinol. 27:219–240. doi:10.1016/j.domaniend.2004.06.007. 15451071

[jkab424-B60] Ou J , Liu H, Yu J, Kelliher MA, Castilla LH, et al 2018. ATAC-SeqQC: a bioconductor package for post-alignment quality assessment of ATAC-seq data. BMC Genomics. 19:1–13. doi:10.1186/s12864-018-4559-3. 29490630 PMC5831847

[jkab424-B61] Pai AA , Pritchard JK, Gilad Y. 2015. The genetic and mechanistic basis for variation in gene regulation. PLoS Genet. 11:e1004857. doi:10.1371/journal.pgen.1004857. 25569255 PMC4287341

[jkab424-B62] Pardo CE , Kreuzer M, Bee G. 2013. Effect of average litter weight in pigs on growth performance, carcass characteristics and meat quality of the offspring as depending on birth weight. Animal. 7:1884–1892. doi:10.1017/S1751731113001419. 23896082

[jkab424-B63] Quinlan AR , Hall IM. 2010. BEDTools: a flexible suite of utilities for comparing genomic features. Bioinformatics. 26:841–842. doi:10.1093/bioinformatics/btq033. 20110278 PMC2832824

[jkab424-B64] R Core Team. 2017. R: A Language and Environment for Statistical Computing. R Found Stat Comput. https://www.r-project.org/.

[jkab424-B65] Ramírez F , Ryan DP, Grüning B, Bhardwaj V, Kilpert F, et al 2016. deepTools2: a next generation web server for deep-sequencing data analysis. Nucleic Acids Res. 44: W160–W165. doi:10.1093/nar/gkw257. 27079975 PMC4987876

[jkab424-B66] Rehfeldt C , Kuhn G. 2006. Consequences of birth weight for postnatal growth performance and carcass quality in pigs as related to myogenesis. J Anim Sci. 84: E113–E123. doi:10.2527/2006.8413_supplE113x. 16582082

[jkab424-B67] Roelofs S , Godding L, de Haan JR, van der Staay FJ, Nordquist RE. 2019. Effects of parity and litter size on cortisol measures in commercially housed sows and their offspring. Physiol Behav. 201:83–90. doi:10.1016/j.physbeh.2018.12.014. 30553897

[jkab424-B68] Rudar M , Fiorotto ML, Davis TA. 2019. Regulation of muscle growth in early postnatal life in a swine model. Annu Rev Anim Biosci. 7:309–335. doi:10.1146/annurev-animal-020518-115130. 30388025 PMC7032524

[jkab424-B69] Salavati. 2021. Code repository for the study “*Profiling of open chromatin in developing pig (Sus scrofa) muscle to identify regulatory regions*”. https://msalavat@bitbucket.org/msalavat/pig_muscle.git. (Accessed: 2021 October 29). https://bitbucket.org/msalavat/pig_muscle. 10.1093/g3journal/jkab424 PMC9210303 34897420

[jkab424-B70] Soneson C , Love MI, Robinson MD. 2016. Differential analyses for RNA-seq: transcript-level estimates improve gene-level inferences. F1000 Research 2016, 4:1521. doi:10.12688/F1000RESEARCH.7563.2. PMC4712774 26925227

[jkab424-B71] Stange K , Miersch C, Sponder G, Röntgen M. 2020. Low birth weight influences the postnatal abundance and characteristics of satellite cell subpopulations in pigs. Sci Rep. 10:6149. doi:10.1038/s41598-020-62779-1. 32273524 PMC7145795

[jkab424-B72] Stenhouse C , Hogg CO, Ashworth CJ. 2018. Associations between fetal size, sex and both proliferation and apoptosis at the porcine feto-maternal interface. Placenta. 70:15–24. doi:10.1016/j.placenta.2018.08.006. 30316322 PMC6215148

[jkab424-B73] Summers KM , Bush SJ, Wu C, Su AI, Muriuki C, et al 2020. Functional annotation of the transcriptome of the pig, *Sus scrofa*, based upon network analysis of an RNA-Seq transcriptional atlas. Front Genet. 10:1355. doi:10.3389/fgene.2019.01355. 32117413 PMC7034361

[jkab424-B74] Thurman RE , Rynes E, Humbert R, Vierstra J, Maurano MT, et al 2012. The accessible chromatin landscape of the human genome. Nature. 489:75–82. doi:10.1038/nature11232. 22955617 PMC3721348

[jkab424-B75] UniProt CT. 2021. Myogenin. UniProt Consortium European Bioinforma Institute Protein Inf Resour Swiss Inst Bioinforma. https://www.uniprot.org/uniprot/P49812.

[jkab424-B76] Venables WN , Ripley BD. 2002. Modern Applied Statistics with S-Plus. 4th ed. New York: Springer. http://www.stats.ox.ac.uk/pub/MASS4/.

[jkab424-B77] Wang X , Liu X, Deng D, Yu M, Li X. 2016. Genetic determinants of pig birth weight variability. BMC Genet. 17:S15. doi:10.1186/s12863-015-0309-6. PMC4895286 26822294

[jkab424-B78] Warr A , Affara N, Aken B, Beiki H, Bickhart DM, et al 2020. An improved pig reference genome sequence to enable pig genetics and genomics research. Gigascience. 9:1–14. doi:10.1093/gigascience/giaa051. PMC7448572 32543654

[jkab424-B79] Weatherall EL , Avilkina V, Cortes-Araya Y, Dan-Jumbo S, Stenhouse C, et al 2020. Differentiation potential of mesenchymal stem/stromal cells is altered by intrauterine growth restriction. Front Vet Sci. 7:809. 10.3389/fvets.2020.558905 PMC7676910 33251256

[jkab424-B80] Weber FE , Vaughan KT, Reinach FC, Fischman DA. 1993. Complete sequence of human fast‐type and slow‐type muscle myosin‐binding‐protein C (*MyBP‐C*): differential expression, conserved domain structure and chromosome assignment. Eur J Biochem. 216:661–669. doi:10.1111/j.1432-1033.1993.tb18186.x. 8375400

[jkab424-B81] Wigmore PM , Stickland NC. 1983. Muscle development in large and small pig fetuses. J Anat. 137(Pt 2):235–245. 6630038 PMC1171817

[jkab424-B82] Wu J , Xu J, Liu B, Yao G, Wang P, et al 2018. Chromatin analysis in human early development reveals epigenetic transition during ZGA. Nature. 557:256–260. doi:10.1038/s41586-018-0080-8. 29720659

[jkab424-B83] Xue H-LL , Zhou Z-XX. 2006. Effects of the *MyoG* gene on the partial growth traits in pigs. Acta Genet Sin. 33:992–997. doi:10.1016/S0379-4172(06)60134-0. 17112970

[jkab424-B84] Yan F , Powell DR, Curtis DJ, Wong NC. 2020. From reads to insight: a Hitchhiker’s guide to ATAC-seq data analysis. Genome Biol. 21:22. doi:10.1186/s13059-020-1929-3. 32014034 PMC6996192

[jkab424-B85] Yan L , Guo H, Hu B, Li R, Yong J, et al 2016. Epigenomic landscape of human fetal brain, heart, and liver. J Biol Chem. 291:4386–4398. doi:10.1074/jbc.M115.672931. 26719341 PMC4813467

[jkab424-B86] Yang XR , Yu B, Mao XB, Zheng P, He J, et al 2015. Lean and obese pig breeds exhibit differences in prenatal gene expression profiles of muscle development. Animal. 9:28–34. doi:10.1017/S1751731114002316. 25229314

[jkab424-B87] Yu G , Wang LG, He QY. 2015. ChIP seeker: an R/bioconductor package for ChIP peak annotation, comparison and visualization. Bioinformatics. 31:2382–2383. doi:10.1093/bioinformatics/btv145. (Accessed: 2020 July 20). http://www.bioconductor.org/packages/release/bioc/html/ChIPseeker.html. 25765347

[jkab424-B88] Yue J , Hou X, Liu X, Ligang W, Gao H, et al 2021. The landscape of chromatin accessibility in skeletal muscle during embryonic development in pigs. J Anim Sci Biotechnol. 12:1–15. doi:10.1186/s40104-021-00577-z. 33934724 PMC8091695

[jkab424-B89] Zhao X , Mo D, Li A, Gong W, Xiao S, et al 2011. Comparative analyses by sequencing of transcriptomes during skeletal muscle development between pig breeds differing in muscle growth rate and fatness. PLoS One. 6:e19774. 21637832 10.1371/journal.pone.0019774 PMC3102668

[jkab424-B90] Zhao Y , Li J, Liu H, Xi Y, Xue M, et al 2015. Dynamic transcriptome profiles of skeletal muscle tissue across 11 developmental stages for both Tongcheng and Yorkshire pigs. BMC Genomics. 16:377. doi:10.1186/s12864-015-1580-7. 25962502 PMC4437458

