# Supplementary figures and images for: Profiling of open chromatin in developing pig (Sus scrofa) muscle to identify regulatory regions
Source: G3 (Bethesda). 2021 Dec 13;12(2):jkab424. doi: 10.1093/g3journal/jkab424 (PMC9210303; doi:10.1093/g3journal/jkab424)

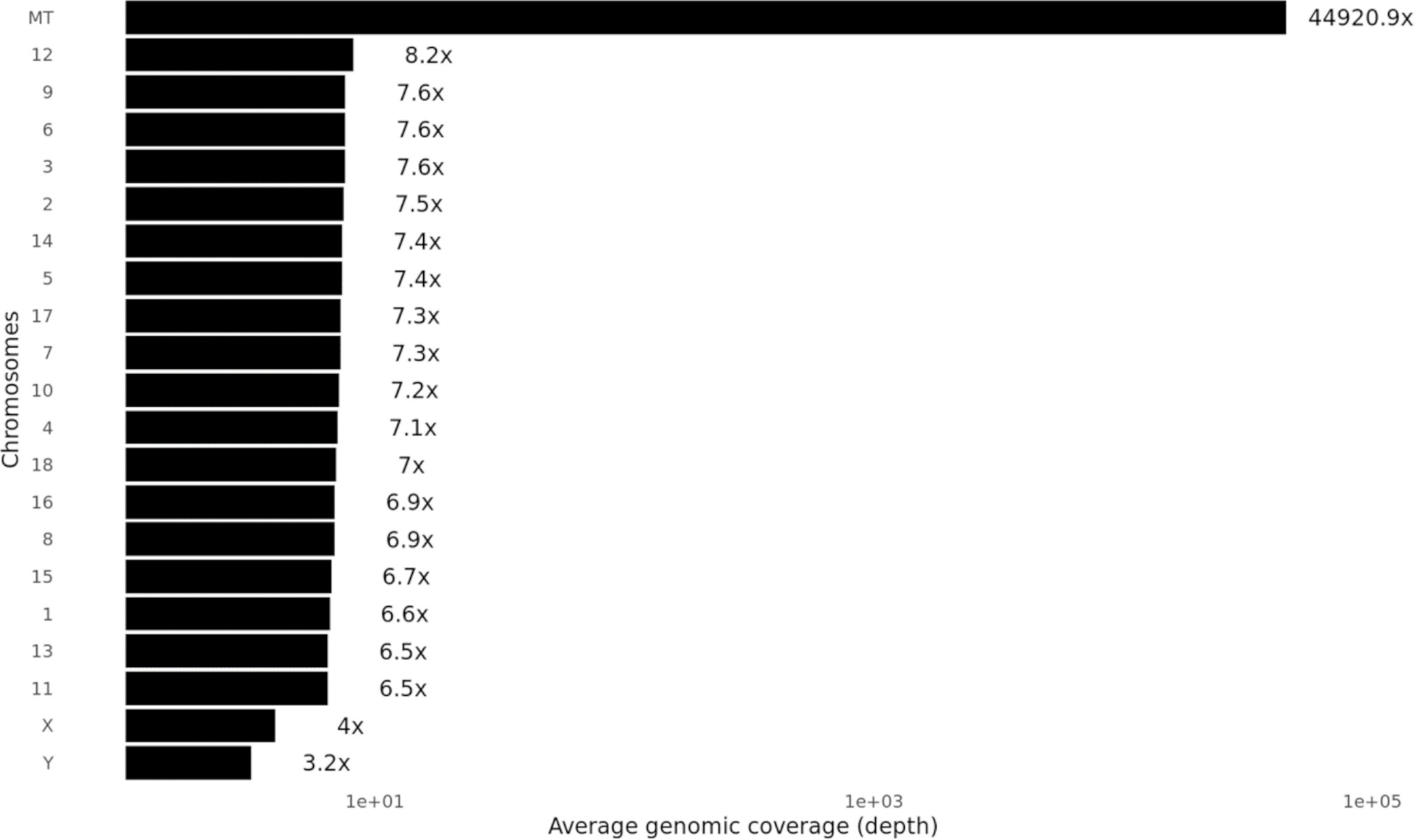

Supplement: jkab424_Supplemental_Figure_S1 [file jkab424_supplemental_figure_s1.jpeg]

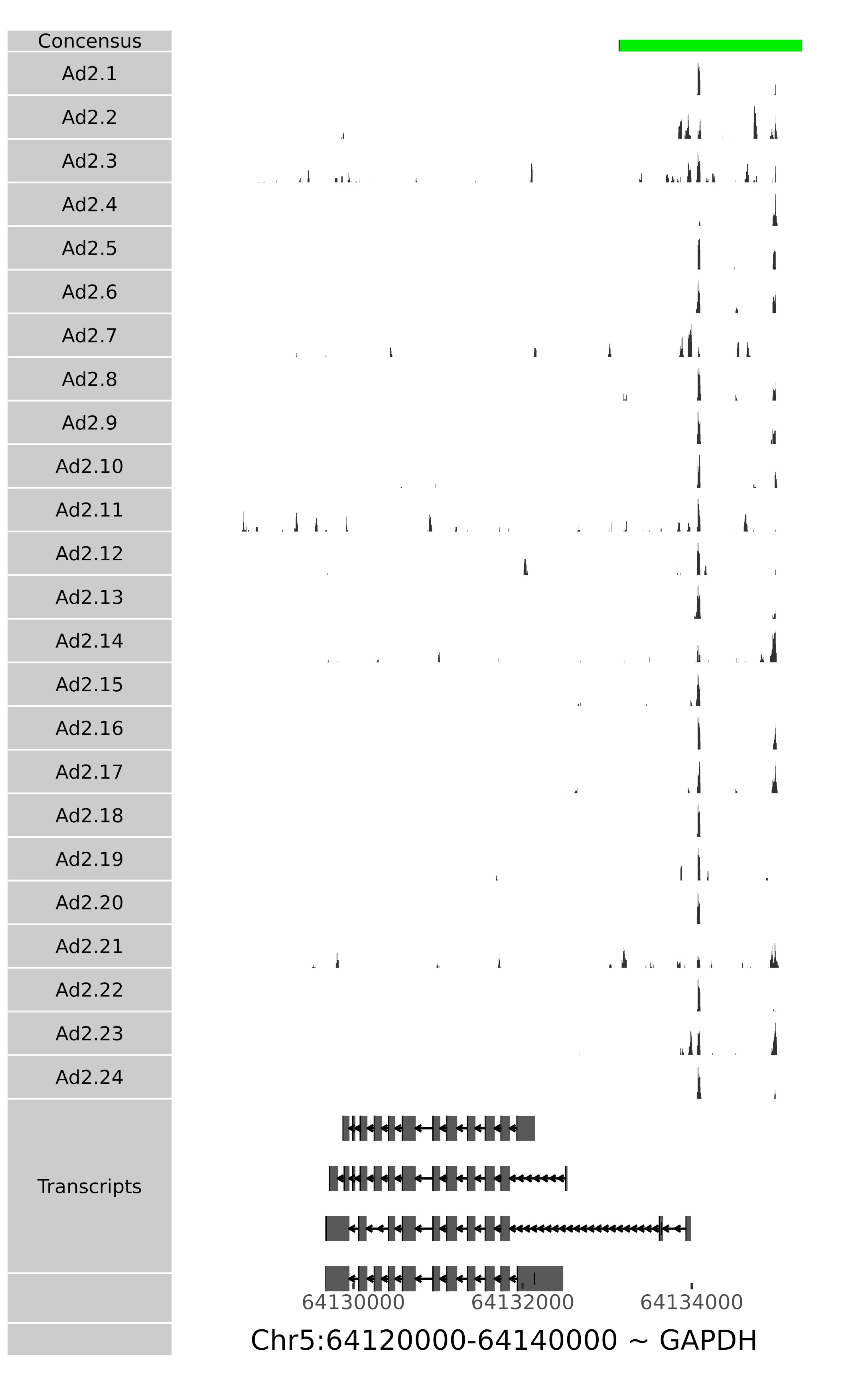

Supplement: jkab424_Supplemental_Figure_S2 [file jkab424_supplemental_figure_s2.jpeg]

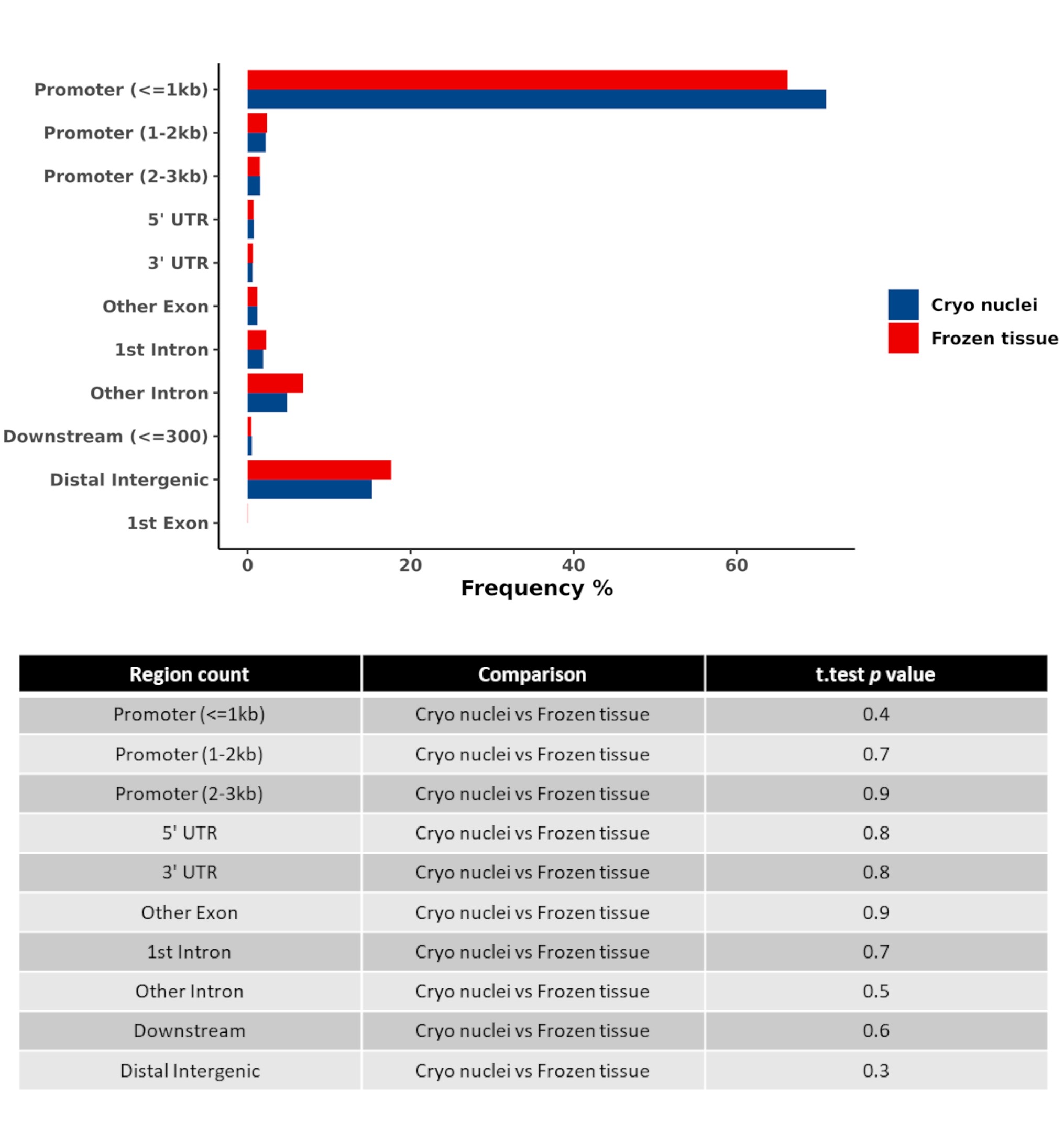

Supplement: jkab424_Supplemental_Figure_S3 [file jkab424_supplemental_figure_s3.jpeg]

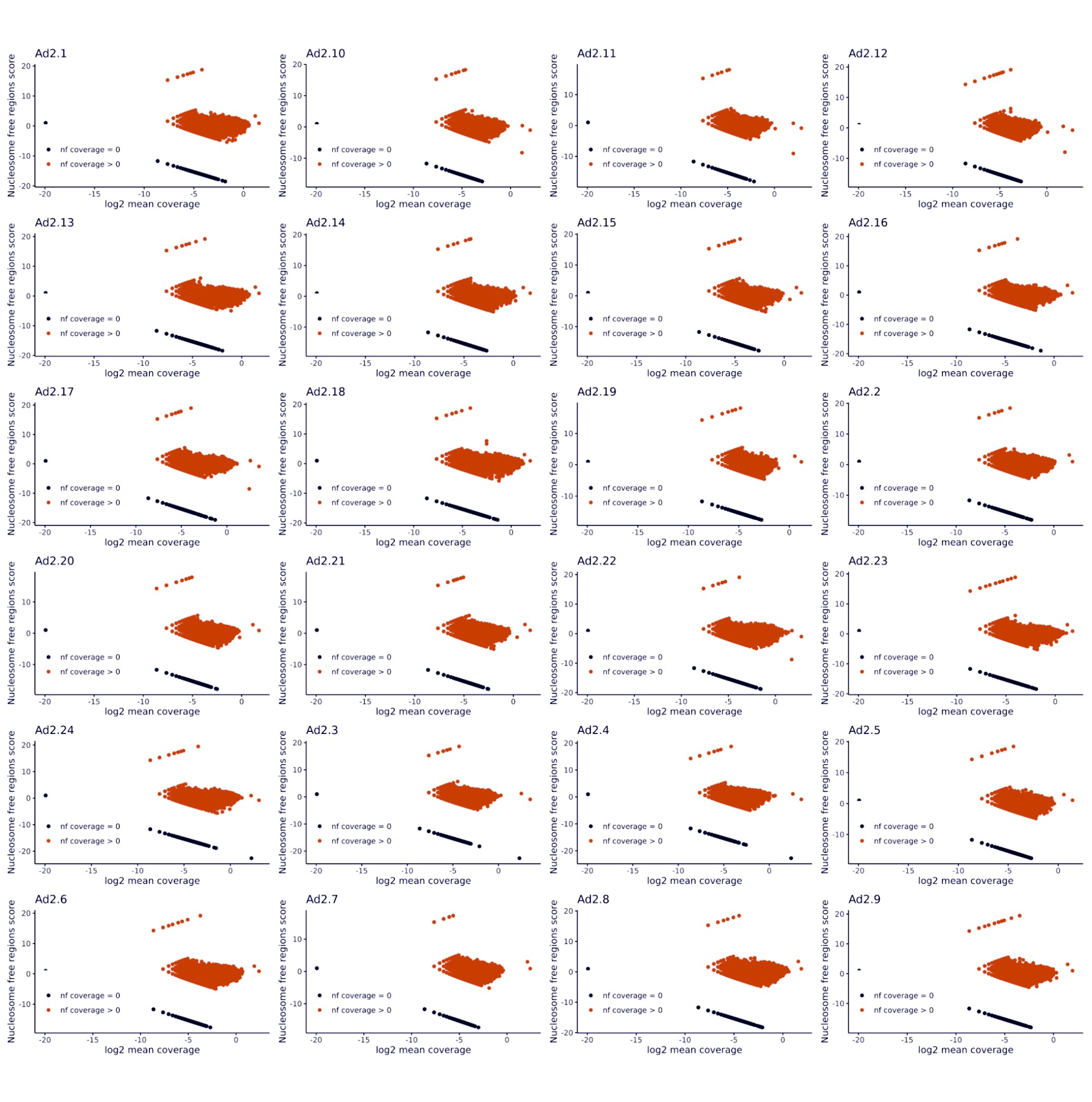

Supplement: jkab424_Supplemental_Figure_S4 [file jkab424_supplemental_figure_s4.jpeg]
