# Supplementary material for: Profiling of open chromatin in developing pig (Sus scrofa) muscle to identify regulatory regions
Source: G3 (Bethesda). 2021 Dec 13;12(2):jkab424. doi: 10.1093/g3journal/jkab424 (PMC9210303; doi:10.1093/g3journal/jkab424)
Supplement: jkab424_Supplemental_Material_Legends [file jkab424_supplemental_material_legends.docx]

**Supplemental Material**

Supplementary Table 1: Weights and crown-rump lengths of all piglets included in the study.

Supplementary Table 2: Details of components of all buffers used for ATAC‑Seq sample collection and library preparation.

Supplementary Table 3: Details of the primers (Ad2.x variable index) used for generating each ATAC‑Seq library. Each sample is barcoded with a different variable index.

Supplementary Table 4: Summary table of RNA‑Sequencing and ATAC‑Sequencing quality control metrics for all samples. 'N/A' indicates that sequencing did not occur for that sample.

Supplementary Table 5: Results of differential expression analysis of large vs average vs small piglet sizes at Day 90 of gestation using a DESeq2 glm model. Only genes with FDR < 0.1 have been included.

Supplementary Table 6: QC metrics recommended by the ENCODE for all 24 ATAC-Seq libraries.

Supplementary_file_S1.zip: The collection of scripts and BED files corresponding to the ATAC‑Seq peak coordinates for the sizes of piglets at Day 90.

Supplementary_file_S2.zip: The library preparation metadata and sequencing read depth metrics of the ATAC‑Seq samples (n=24).

Supplementary_file_S3.zip: The differential peak analysis outputs for timeseries and Day 90 piglet size comparisons.

Supplementary Figure S1: Average genomic coverage of the ATAC‑Seq dataset by chromosome.

Supplementary Figure S2: Genomic track visualisation of the ATAC‑Seq dataset by raw read coverage at the gene coordinates for *GAPDH for each sample individually.*

Supplementary Figure S3: Metrics to compare ATAC‑Seq peaks in libraries prepared from either cryopreserved nuclei from fresh tissue (blue) or from flash frozen tissue (red). Both sets of samples were collected from the semitendinosus muscle from the same six-week-old piglets. The results of a t-test comparison of the protocols for each of the genomic feature categories is presented in the enclosed table.

Supplementary Figure S4: Scatter plots of the Nucleosome free region score (NFR-score) flanking TSSs in all ATAC-Seq libraries (n=24). The Sscrofa 11.1 transcript models were used for calculating NFR score flanking TSSs in ATAC-SeqQC v1.14.4 package in R. The 150bp upstream counts (n1), nucleosome free region 100bp counts (nf) and 150bp downstream counts (n2) were used to calculate the NFR-score using the following equation:

$$NFRscore= log2\left( nf \right)-log2(\frac{n1+n2}{2})$$

The nucleosome free (middle 100bp of the TSS window) score of 0 is coloured differently (blue) from regions with nf score > 0 (orange).
